# *Drosophila* Dpp and BMP signaling directly regulates *dpp* transcription for optimal ligand production

**DOI:** 10.1242/dev.204488

**Published:** 2025-11-10

**Authors:** Yan Zhang, Ying Feng, Xiaolei Ye, Yi Lin, Ling Zeng, Yuting Luo, Lin Zhou, Chenghao Shen, Weiqi Hu, Dong Yan, Xinhua Lin

**Affiliations:** ^1^ State Key Laboratory of Eye Health, Eye Hospital, Wenzhou Medical University, Wenzhou, Zhejiang 325027, China; ^2^ Oujiang Laboratory (Zhejiang Lab for Regenerative Medicine, Vision and Brain Health), Wenzhou, Zhejiang 325000, China; ^3^ Zhejiang Key Laboratory of Key Technologies for Visual Pathway Reconstruction, Eye Hospital, Wenzhou Medical University, Wenzhou, Zhejiang 325027, China; ^4^ State Key Laboratory of Genetic and Development of Complex Phenotypes, School of Life Sciences, Fudan University, Shanghai 200438, China; ^5^ Greater Bay Area Institute of Precision Medicine (Guangzhou), Zhongshan Hospital, Fudan University, Shanghai 200438, China

**Keywords:** *Drosophila melanogaster*, Dpp/BMP signaling, *Decapentaplegic* (*dpp*), Negative feedback, Ligand dosage, Morphogen gradient

## Abstract

Precise control of morphogen dosage in the source is essential for establishing developmental patterning and maintaining signaling stability. Here, we demonstrate that in *Drosophila* wing imaginal discs Decapentaplegic and BMP (Dpp/BMP) signaling modulates expression of the ligand Dpp through direct transcriptional auto-regulation. The results show that reduction of Dpp/BMP signaling upregulates *dpp* transcription, whereas enhanced signaling represses it. This transcriptional negative-feedback mechanism primarily depends on the BMP-silencer-element (BMP-SE) complex binding to *BMP-SE* motifs at the *dpp* downstream distal cis-regulatory region. We validated this mechanism through BMP-SE complex interference and *BMP-SE* motif disruption assays. Our findings extend the morphogen source-sink theory by uncovering that the Dpp/BMP signaling pathway dynamically balances Dpp dosage via transcriptional auto-modulation, thereby ensuring optimal ligand production for signaling homeostasis.

## Teaser

**Summary:** In *Drosophila* wing imaginal discs, Dpp and BMP signaling modulates expression of the Dpp through direct transcriptional auto-regulation.

## INTRODUCTION

The Bone Morphogenetic Protein (BMP) signaling pathway, initially identified in the study of vertebrate bone and cartilage (Thielen et al., 2019), is conserved across various model organisms and participates in diverse biological processes. In *Drosophila*, the BMP pathway is known as the Dpp (Decapentaplegic) pathway, named after the primary BMP ligand-encoding gene *dpp* (Spencer et al., 1982)*.* During embryonic development, *dpp* is essential for establishing dorsal-ventral polarity, segmentation, compartment boundaries (Ferguson and Anderson, 1992) and mesoderm formation (Lee and Frasch, 2005). It also plays roles in tissue growth (Akiyama and Gibson, 2015), trachea formation (Ribeiro et al., 2002; Roy et al., 2014), germ cell regulation (Xie and Spradling, 1998; Kawase et al., 2004), nerve development (Allan et al., 2003), intestinal stem cell regulation (Tian and Jiang, 2014) and other important biological events.

During Dpp and BMP (Dpp/BMP) signal transduction, the immature Dpp ligand is cleaved by the Furin convertase in the Golgi apparatus, then the mature peptides are secreted and become functional (Kunnapuu et al., 2009, 2014). BMP receptors, which belong to the TGFβ serine/threonine kinase family, consist of type I and type II subtypes. Type I receptors include Saxophone (Sax) (Neul and Ferguson, 1998) and Thickveins (Tkv) (Nguyen et al., 1998), while the type II receptor is known as Punt (Put) (Letsou et al., 1995). In some contexts, Wit can also functions as a BMP type II receptor (McCabe et al., 2003; Rawson et al., 2003; Anderson and Wharton, 2017). These receptors mediate signal transduction through a series of phosphorylation cascades (Chen et al., 1998).

The Dpp/BMP transcriptional factor Mad (Mothers against *dpp*) (Kim et al., 1997) is analogous to Receptor-regulated-Smad (R-Smad) in vertebrates. Medea (Med) (Das et al., 1998; Wisotzkey et al., 1998) is the homolog of vertebrate Smad4, also known as the Common-Smad (Co-Smad). Upon signaling activation, the pMad/Med heterotrimer (Smad complex) collaborates with other transcription factors to activate or suppress transcription of target genes (Gao et al., 2005; Chacko et al., 2004). In the absence of Co-Smad, a single R-Smad exhibits weak DNA-binding ability (BabuRajendran et al., 2010).

Among co-factors, Schnurri (Shn), a zinc-finger C2H2 transcription factor, is a key component of the BMP-SE complex (Udagawa et al., 2000; Marty et al., 2000). Shn acts as an inhibitory co-factor recruited by the pMad/Med (Smad) complex, when it binds to *BMP-SE* motifs (Pyrowolakis et al., 2004; Charbonnier et al., 2015). The Smad complex binds independently to both *BMP-SE* and *BMP-Activating-Element* (*BMP-AE*). However, its conformation differs between motifs, determining the recruitment of distinct co-factors. On *BMP-SE*, the Smad complex recruits Shn, and the Mad/Med/Shn complex further recruits other repressive cofactors, whereas, on *BMP-AE*, it recruits activating co-factors (Weiss et al., 2010; Chayengia et al., 2019). Notably, the transcriptional repressor Brinker (Brk) antagonizes Dpp signaling at *BMP-AE* sites by competing with the Smad complex, thereby inhibiting target genes transcription (Minami et al., 1999; Muller et al., 2003). In addition, other factors such as Smad on X (Smox) (Takaesu et al., 2006) and the NFκB-like protein Relish (Rel) (Shokri et al., 2019; Yao et al., 2006) have also been reported to participate in Smad-associated regulatory complexes, further underscoring the complexity of Dpp/BMP signaling regulation.

Dpp/BMP signaling strength can be modulated through several layers of feedback regulation. Eldar et al. proposed the robustness theory for the BMP pathway, suggesting it maintains signaling intensity through various regulatory mechanisms (Eldar et al., 2002). Pathway stability is regulated by multiple self-targets and other regulated genes, including *daughters against dpp* (*dad*) (Tsuneizumi et al., 1997; Ogiso et al., 2011), *tkv* (Haerry et al., 1998), *brk* (Minami et al., 1999; Winter and Campbell, 2004), *pentagone* (*pent*) (Hamaratoglu et al., 2011) and *nord* (Akiyama et al., 2022), among others. According to the morphogen source-sink theory, gradients are established through a balance between localized production (source), and distributed degradation, sequestration and consumption (sink) (Eldar et al., 2002; Vuilleumier et al., 2010; Zhu et al., 2020; Wartlick et al., 2011). In the *Drosophila* wing imaginal disc, Dpp ligand is produced in a spatially restricted manner and forms a gradient that patterns tissue development (Nellen et al., 1996; Minami et al., 1999). The source-sink balance contributes to signaling robustness and ensures precise local signaling strength (Eldar et al., 2002, 2004). Thus, the ligand production regulation at the source is crucial.

Although our findings represent the first analysis of *dpp* transcriptional autoregulation via self-feedback, related phenomena have been described previously without detailed mechanistic explanation (Haerry et al., 1998; Hepker et al., 1999; Akiyama et al., 2022). Here, our findings reveal that Dpp/BMP signaling directly modulates *dpp* transcription within a feedback loop, primarily mediated through the *BMP-SE*-dependent repression at the *dpp_BS 3.0-plus* cisregulatory region. This autoregulated Dpp expression ensures a stable ligand source for the Dpp/BMP signaling pathway.

## RESULTS

### *dpp* genetic engineering alleles and associated strategies

**Fig. 1.**
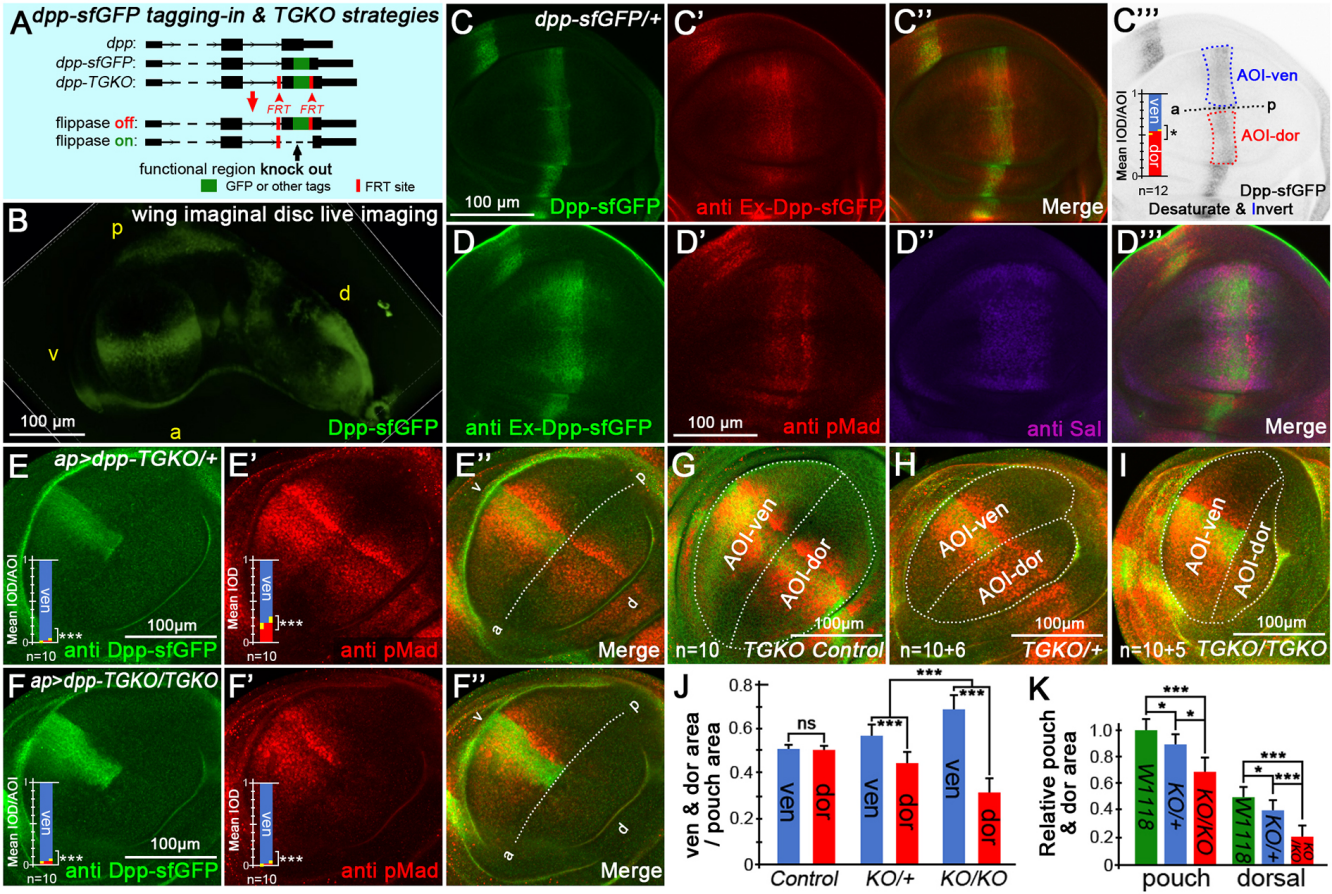
**Genetic engineering strategies for *dpp* tagging in strains and TGKO system.** (A) Genetic engineering strategy for sfGFP tagging strain and a diagrammatic sketch of the TGKO conditional knockout strategy. (B) Live image of Dpp-sfGFP in the third instar larval wing imaginal disc. a, anterior; d, dorsal; p, posterior; v, ventral. (C) Original sfGFP fluorescence, (C′) extracellular staining of Dpp-sfGFP, (C″) merge and (C‴) statistical analysis of sfGFP fluorescence. (D) Extracellular staining of Dpp-sfGFP in another wing disc, (D′) staining of the *Drosophila* Dpp/BMP signaling target pMad, (D″) staining of another target, Sal, and (D‴) merge. (E-E″) Heterozygous knockout of endogenous *dpp-sfGFP* using the TGKO system, combined with pMad staining. (F-F″) Homozygous knockout of *dpp-sfGFP* using the TGKO system, combined with pMad staining. (G-I) Division of ventral and dorsal regions in the wing disc pouch. The sample *n* in statistical analyses are combined with the Fig. S5 sample of Sal staining, shown as n1(pMad)+n2(Sal). (J) Relative area changes in samples (ventral or dorsal/pouch). (K) Relative area changes between groups (pouch/pouch and dorsal/pouch).

We found that the one-step tagging in strategy (Yu et al., 2021) is suitable for the haploid lethal gene *dpp*. The superfolder GFP (sfGFP)-encoding sequence was inserted into an exon of the endogenous *dpp* locus (G464/G465, Fig. 1A and Fig. S1A). The GFP fluorescence was directly observable but weak without staining, primarily representing both pro-Dpp and mature Dpp forms; partial untagged Dpp might exist in this allele (Fig. S1B,C), and the mixed Dpp pool is potentially beneficial to survival (Fig. S2). In the Dpp-producing region, Dpp-sfGFP levels were slightly higher in the dorsal region than in the ventral region (Fig. 1C‴). The extracellular Dpp-sfGFP diffusion pattern differs from that of the *dpp-Gal4/UAS-dpp-GFP* binary system (Belenkaya et al., 2004); the former tends to diffuse anteriorly but not throughout the entire pouch compared to latter one (Fig. 1B and Movie 1). This asymmetry may arise from uneven distribution of the receptor Tkv (Figs S10B′ and S11C′) and ligand endocytosis, consistent with a recent finding (Matsuda et al., 2021). Extracellular Dpp-sfGFP was stained using a designated protocol (Strigini and Cohen, 2000), revealing a broader diffuse range (Fig. 1C′,D). Signaling activity was monitored via pMad and Spalt (Sal), which were co-stained with extracellular Dpp-sfGFP (Fig. 1D′,D″). Furthermore, the endogenous Dpp-sfGFP expression patterns in stage 14 embryos, brains and eye imaginal discs are shown in Fig. S3. We compared ligand activity between wild-type *dpp*, heterozygous *dpp-sfGFP* and homozygous *dpp-sfGFP* strains (Fig. S4), and concluded that this *dpp-sfGFP* allele is weaker in signaling activation than wild-type *dpp*.

To directly visualize Dpp during loss-of-function studies, we developed Tag-fused Gene Conditional Knockout (TGKO), an efficient and visible gene knockout strategy. We first generated a sfGFP-tagged fly line with a pair of FRT sites inserted in both flanks of the knockout region (Fig. 1A). Next, we acquired the crossed offspring with the genotype containing: *dpp-TGKO*, *specific-Gal4*, *Gal80^ts^* and *UAS-flippase*, which underwent FRT-mediated excision of the intervening sequence. Knockout efficiency was assessable via tag fluorescence. In *dpp-TGKO* heterozygotes, pMad staining showed moderate reduction (Fig. 1E′). The width of Sal expression became narrow, but without intensity loss over a short range (Fig. S5B‴,C″). Notably, homozygous *dpp-TGKO* nearly eliminated pMad (Fig. 1F′) and Sal (Fig. S5D″) staining in the dorsal pouch, aligning with earlier reports of and essential role for *dpp* in wing disc growth (Akiyama et al., 2022). Dorsal and entire pouch size reduction was pronounced in heterozygous and homozygous tissues (Fig. 1G-K).

Using a similar one-step protocol, we generated multiple endogenous tagging in strains: *dpp-tdTMT*, *dpp-mRFP-V5*, *tkv-sfGFP* (Figs S10B′ and S11C′), *sax-mRFP* (Fig. S6A), *punt-YFP (Venus)* (Fig. S6B) and *mad-mRFP-V5* (see Fig. 7A,B). These lines were independently designed and differ from the strains published by the Gibson lab (Akiyama and Gibson, 2015), the Vincent lab (Bosch et al., 2017), and the Affolter lab (Matsuda et al., 2021). The construct, primers, gRNAs and injection docking fly information for genetic engineering alleles are described in the supplementary Materials and Methods.

### Dpp/BMP signaling regulates Dpp expression in a negative feedback

**Fig. 2.**
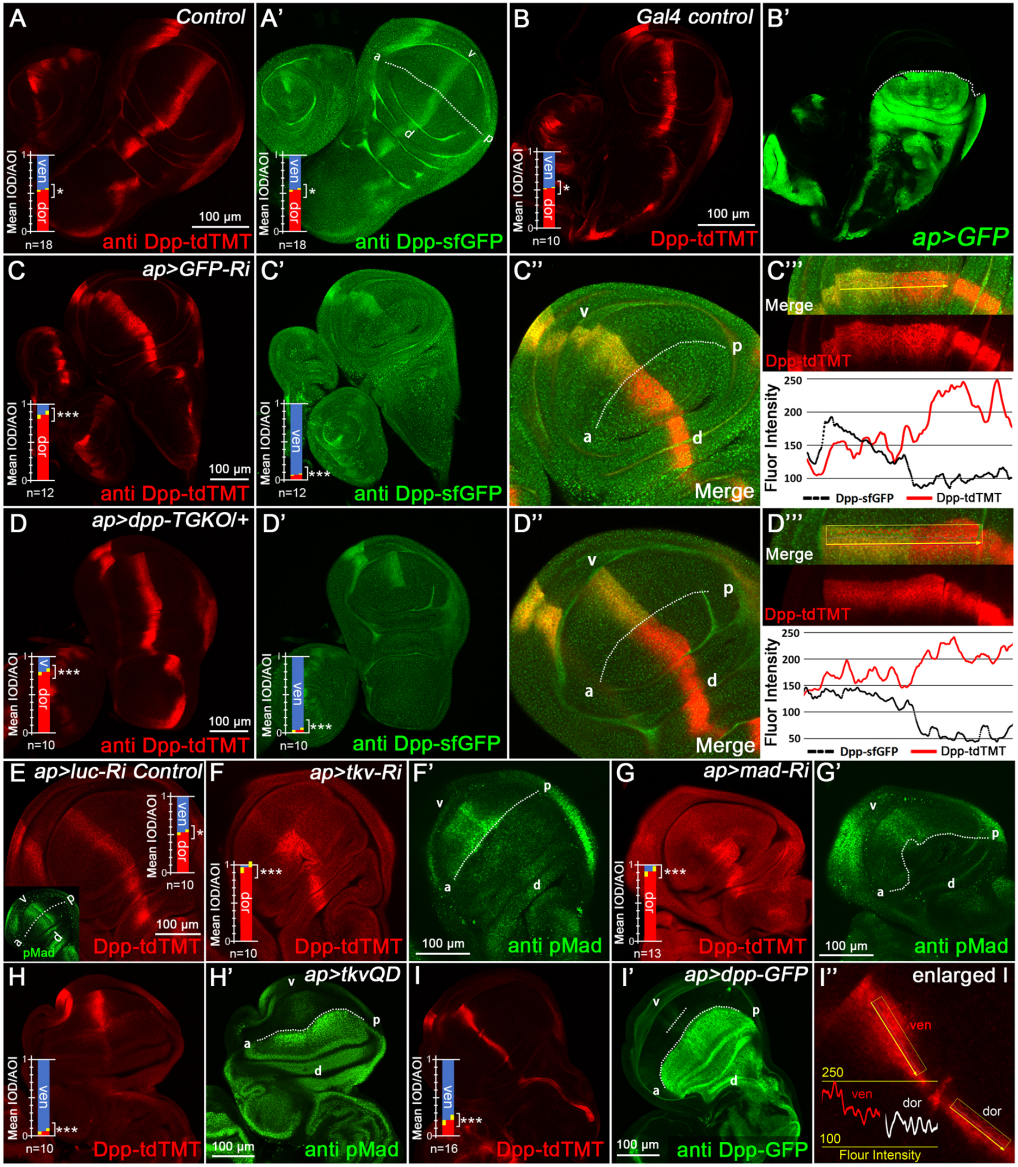
**Dpp/BMP signaling modulates Dpp dose compensation and indicates an existing negative-feedback mechanism.** (A,A′) Staining of Dpp-tdTMT using anti-Tomato (tdTMT) antibody (A) and staining of Dpp-sfGFP with anti-GFP antibody (A′). (B,B′) tdTMT and GFP staining in *ap>GFP* wing discs. (C-C‴) Upon knockdown of Dpp-sfGFP using *ap>GFP-RNAi*, Dpp-tdTMT is significantly increased in the *ap-Gal4* drive region. (D-D‴) Following the *ap>dpp-TGKO* procedure, in contrast to the decrease in Dpp-sfGFP, the Dpp-tdTMT is also markedly increase in the dorsal region. In C‴ and D‴, the black lines represent Dpp-sfGFP level and the red lines represent Dpp-tdTMT level. (E) *ap>luciferase (luc)-RNAi* as a control. (F-G′) Dpp-tdTMT is dramatically upregulated in the dorsal regions of *ap>tkv-RNAi* and *ap>mad-RNAi* pouches. (H-I″) Dpp-tdTMT is significantly decreased in (H,H′) *ap>tkvQD* and (I-I″) *ap>Dpp-GFP* groups dorsal pouch. In I″, *ap>Dpp-GFP* leads to excessive Dpp-GFP diffusion from the dorsal to the ventral region and, thus, significant repression of ventral Dpp-tdTMT expression at a specific distance from the *ap* axis.

To investigate potential crosstalk between *dpp* alleles, we generated multiple fluorescently tagged *dpp* variants (Fig. 2A,A′). Staining confirmed that the widely used *ap-Gal4* does not alter Dpp-tdTMT expression patterns (Fig. 2B,B′). Strikingly, knockdown or knockout of *dpp-sfGFP* via *ap-Gal4>GFP-RNAi* or *ap-Gal4>dpp-TGKO* consistently upregulated Dpp-tdTMT within the *ap-Gal4* expression domain (Fig. 2C-C‴,D-D‴). These results indicate compensatory regulation between *dpp* alleles, where functional reduction in one allele triggers partial compensation by the other within a certain range.

We quantified the compensation efficiency by measuring the changes in the ventral/dorsal fluorescence intensity ratio, which was calculated by mean IOD/AOI (integrated optical density/area of interest). Quantification revealed ∼60% Dpp-tdTMT upregulation in the *GFP-RNAi* group (Fig. 2C) and ∼50% in heterozygous *TGKO* (Fig. 2D). However, monoallelic *dpp* expression failed to fully compensate for gene dosage loss, as evidenced by reduced pMad staining (Fig. 1E′), unrestored Sal expression at long range (Fig. S5B‴,C″) and irreversible size reduction in the dorsal pouch (Fig. 1G-K).

We next probed whether *dpp* autoregulates via its own pathway. Dpp signaling suppression through *tkv* or *mad RNAi* upregulated Dpp-tdTMT (Fig. 2F,G). Conversely, expressing constitutively activated TkvQD (Nellen et al., 1996) and Dpp-GFP, which cell-autonomously (Fig. 2H) or non-autonomously (Fig. 2I,I″) repressed *dpp-tdTMT*. The ventral Dpp expression gradient (Fig. 2I″) indicates that local *dpp* non-autonomously responds to the excess Dpp diffusing from the dorsal side.

Our data demonstrate that *dpp* likely represses its own expression through the Dpp/BMP pathway. This mechanism is conserved in other imaginal discs, such as the eye imaginal disc (Fig. S7B′). However, this mechanism does not function during embryonic development stages. Instead, embryonic *dpp* appears to rely on a feed-forward mechanism, where a weak allele downregulates the expression of another one (Fig. S8). This may be due to the embryonic *dpp* initial transcription and enhanced mechanism may be different. Previous studies have proven that the haploinsufficient (Hin) region is important for embryonic development, while the disc region affects the imaginal discs, and the shortvein (shv) region affects the pupal wing veins (St Johnston et al., 1990; Sotillos and de Celis, 2006). The Hin region does not contain significant Dpp/BMP inhibitory elements (Fig. 6A).

### Dpp/BMP signaling represses *dpp* transcription

**Fig. 3.**
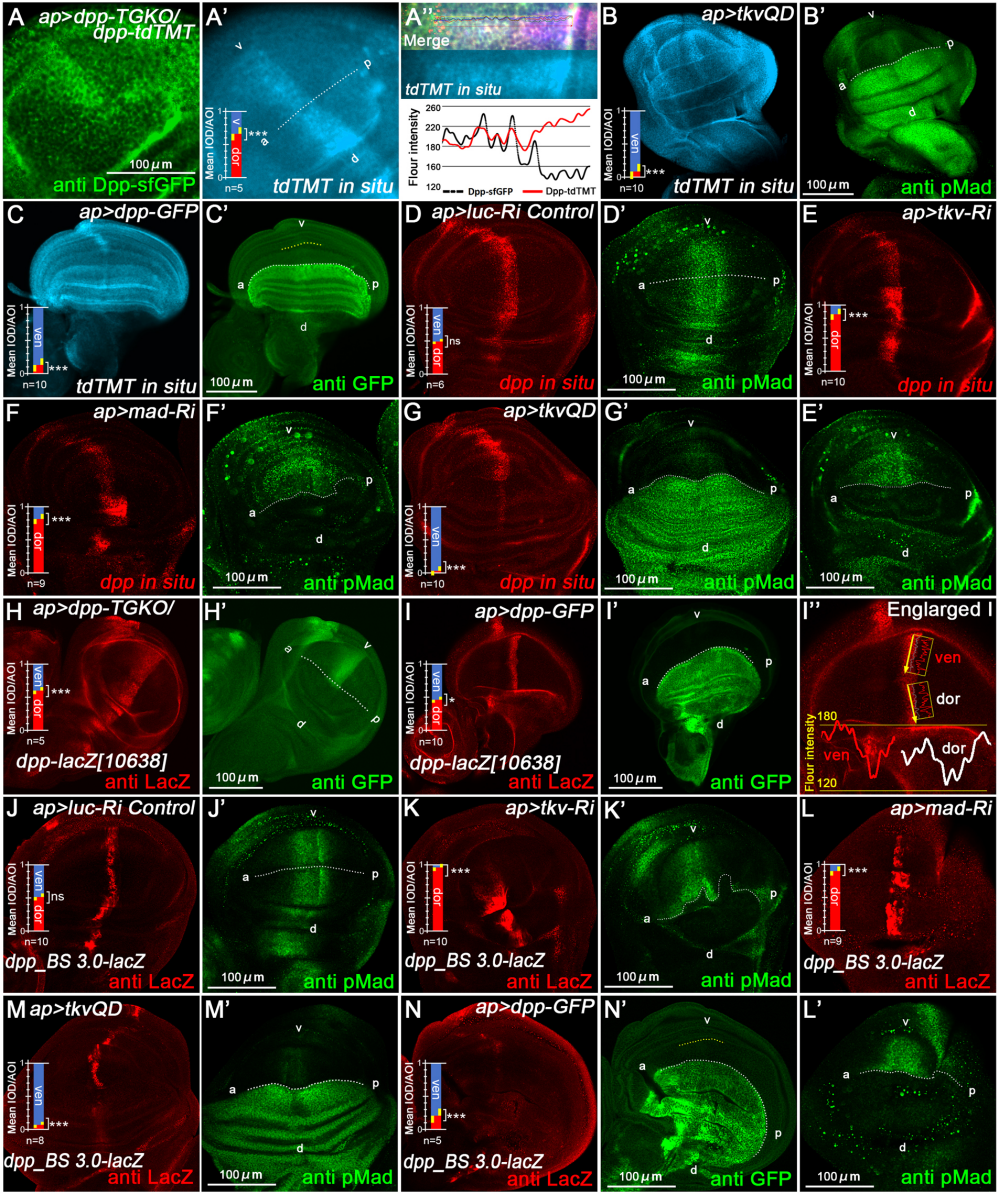
**Dpp/BMP signaling regulates *dpp* mRNA transcription and *lacZ* reporter expression.** (A) Anti-Dpp-sfGFP staining and (A′,A″) tyramide signal amplification (TSA) signal for anti *tdTMT* mRNA *in situ* hybridization in *ap>dpp-TGKO* discs, showing a significant increase in *dpp-sfGFP* knockout regions. In A″, the black line represents Dpp-sfGFP expression levels and the red line represents TSA signals for *tdTMT* mRNA levels. (B,C) TSA signals for the *anti-tdTMT* mRNA probe in *ap>TkvQD* and *ap>Dpp-GFP* discs, showing a sharp reduction in *ap-Gal4* regions. (D-G) TSA signals for the anti*-dpp* mRNA probe and anti-pMad staining (D′-G′) in control (D,D′), *ap>tkv-RNAi* (E,E′), *ap>mad-RNAi* (F,F′) and *ap>tkvQD* (G,G′) discs. (H-I″) Anti-β-galactosidase (anti-LacZ) antibody staining for *dpp-lacZ[10638]* and anti-GFP staining (H′,I′) in *ap>dpp-TGKO* (H,H′) and *ap>Dpp-GFP* (I,I′,I″) discs. (J-N′) Anti-LacZ staining for *dpp-lacZ[BS3.0]*, anti-pMad (J′-M′) and anti-GFP (N′) antibody staining in *ap>luc-RNAi* (control) (J,J′), *ap>tkv-RNAi* (K,K′), *ap>mad-RNAi* (L,L′), *ap>tkvQD* (M,M′) and *ap>Dpp-GFP* (N,N′) discs.

To determine whether feedback occurs at the transcriptional level, we performed *in situ* hybridization assays to detect mRNA of *dpp-tdTMT* and *dpp*. We used a *tdTMT-*specific mRNA probe for the TGKO group and a *dpp*-specific probe for all other groups. Knockout of *dpp-sfGFP* in the dorsal pouch significantly increased *dpp-tdTMT* mRNA levels (Fig. 3A′,A″). Conversely, ectopic expression of TkvQD (Fig. 3B) or Dpp-GFP (Fig. 3C) enhanced Dpp/BMP signaling and dramatically reduced *tdTMT* mRNA signal (Fig. 3B,C). Endogenous *dpp* mRNA hybridization results mirrored those of *dpp-tdTMT*: downregulating Dpp/BMP signaling increased *dpp* mRNA signals (Fig. 3E,F), while upregulation decreased them (Fig. 3G). These *in situ* hybridization results aligned with Dpp protein observations (Fig. 2C-I″), collectively demonstrating transcriptional regulation of *dpp* by its own signaling pathway. RT-qPCR analysis of all four *dpp* isoforms revealed that *dpp-RA*, *RC* and *RE* significantly respond to downregulated Dpp/BMP signaling (Fig. S9A,A′).

In *Drosophila*, enhancer traps and genomic cloning reporter have long served as transcriptional activity reporters. We employed two systems: the enhancer trap strain *dpp-lacZ[10638]* (Spradling et al., 1999; Bellen et al., 2004) with P-element insertion upstream of the *dpp-RE* transcriptional start site (TSS; blue diamond in Fig. 5A), and the genomic cloning reporter *dpp-lacZ [BS_3.0]* (Blackman et al., 1991), containing a 10.4 kb DNA segment start at downstream ∼18 kb from *dpp* (Fig. 5A). *dpp-lacZ [10638]* expression showed minimal changes in the dorsal region upon *dpp-sfGFP* knockout or Dpp/BMP signaling upregulation (Fig. 3H-I″). In contrast, *dpp_BS 3.0-lacZ* expression was markedly altered by Dpp/BMP signaling modulation (Fig. 3J-N′), which consistent with protein and mRNA data, suggesting that *dpp_BS 3.0* region is important to regulation.

### The BMP-SE complex plays a dominant role in repressing *dpp* transcription

**Fig. 4.**
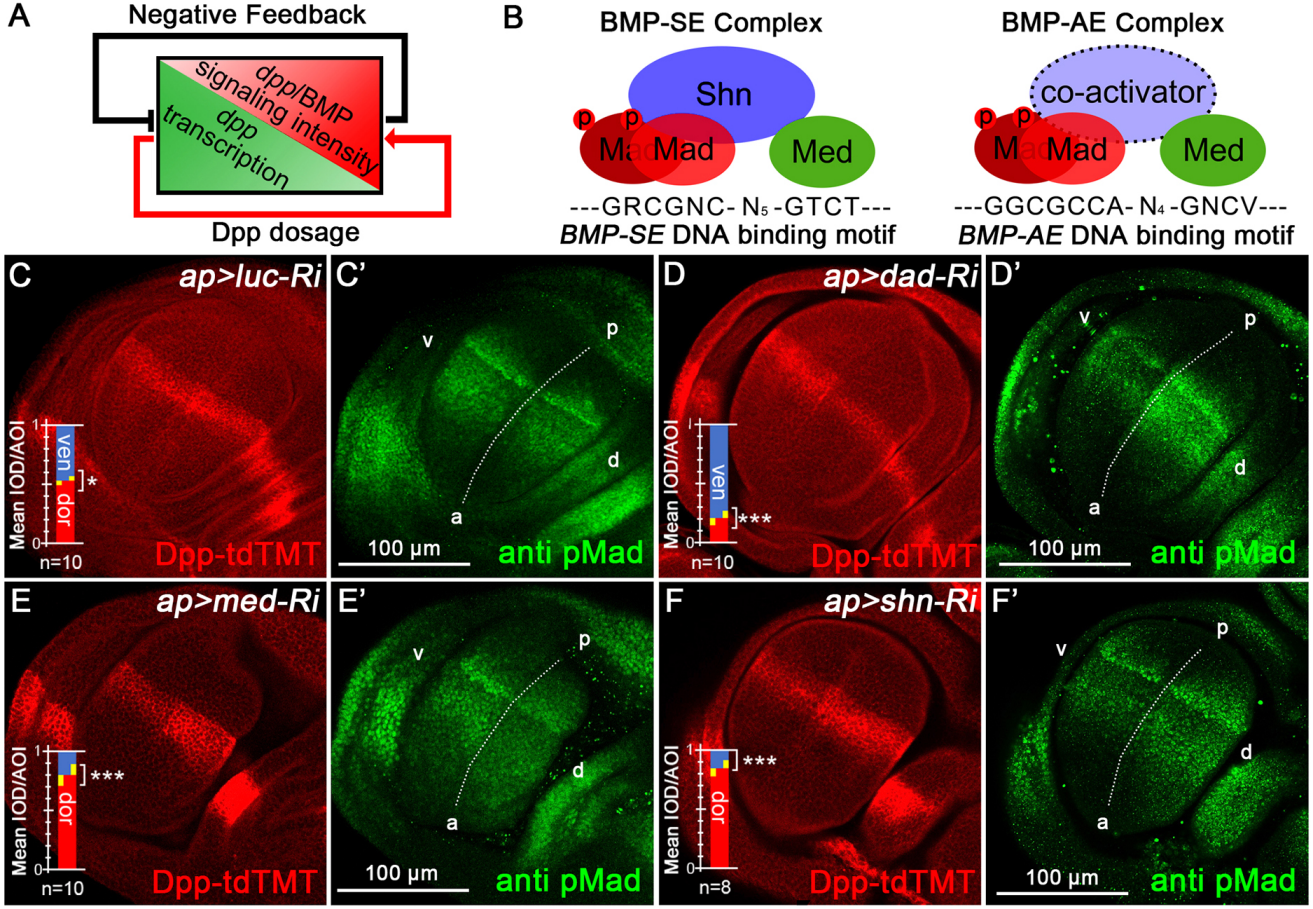
**Disruption of the BMP-SE complex is sufficient to upregulate Dpp expression.** (A) A general model of Dpp/BMP signaling modulating *dpp* transcription in a negative-feedback loop. (B) Schematics of the BMP-SE and BMP-AE complexes with DNA-binding motifs. (C-F′) Dpp-tdTMT and pMad staining in *ap>luc-RNAi* (control) (C,C′) and other BMP-SE complex component knockdown groups (D-F′). Dpp-tdTMT is significantly reduced in (D) the *ap>dad-RNAi* group, but is strongly upregulated in (E) the *ap>med-RNAi* and (F) *ap>shn-RNAi* groups.

This study has demonstrated that *dpp* transcription is negatively regulated by Dpp/BMP signaling (Fig. 4A). Since this regulation occurs at the transcriptional level, we investigated potential BMP-SE complex involvement. The canonical BMP-SE complex comprises pMad, Med and Shn. A schematic of the BMP*-*SE complex with its consensus binding motif *GRCGNC(N)_5_GTCT* (Charbonnier et al., 2015) and the BMP-AE complex with it consensus sequence *GGCGCCA(N)_4_GNCV* (Weiss et al., 2010) are displayed in Fig. 4B.

Since Dad negatively regulates Mad phosphorylation, affecting Smad complex formation and promoting receptor degradation (Ogiso et al., 2011), we introduced *dad-RNAi* to oppose the effects of *mad* knockdown by elevating pMad levels, providing additional validation. Experiments confirmed the regulatory role of Dad: *dad* knockdown upregulated Dpp/BMP signaling (Fig. 4D′) and repressed Dpp-tdTMT expression (Fig. 4D). We next knocked down other BMP-SE complex components, including Med (Fig. 4E) and Shn (Fig. 4F). Results demonstrate that disrupting any Mad/Med/Shn complex component elevates *dpp* expression. We conclude that Dpp/BMP signaling represses *dpp* expression primarily through the BMP-SE complex.

### CRISPR/Cas9-mediated *BMP-SE*-disruption upregulates Dpp expression

**Fig. 5.**
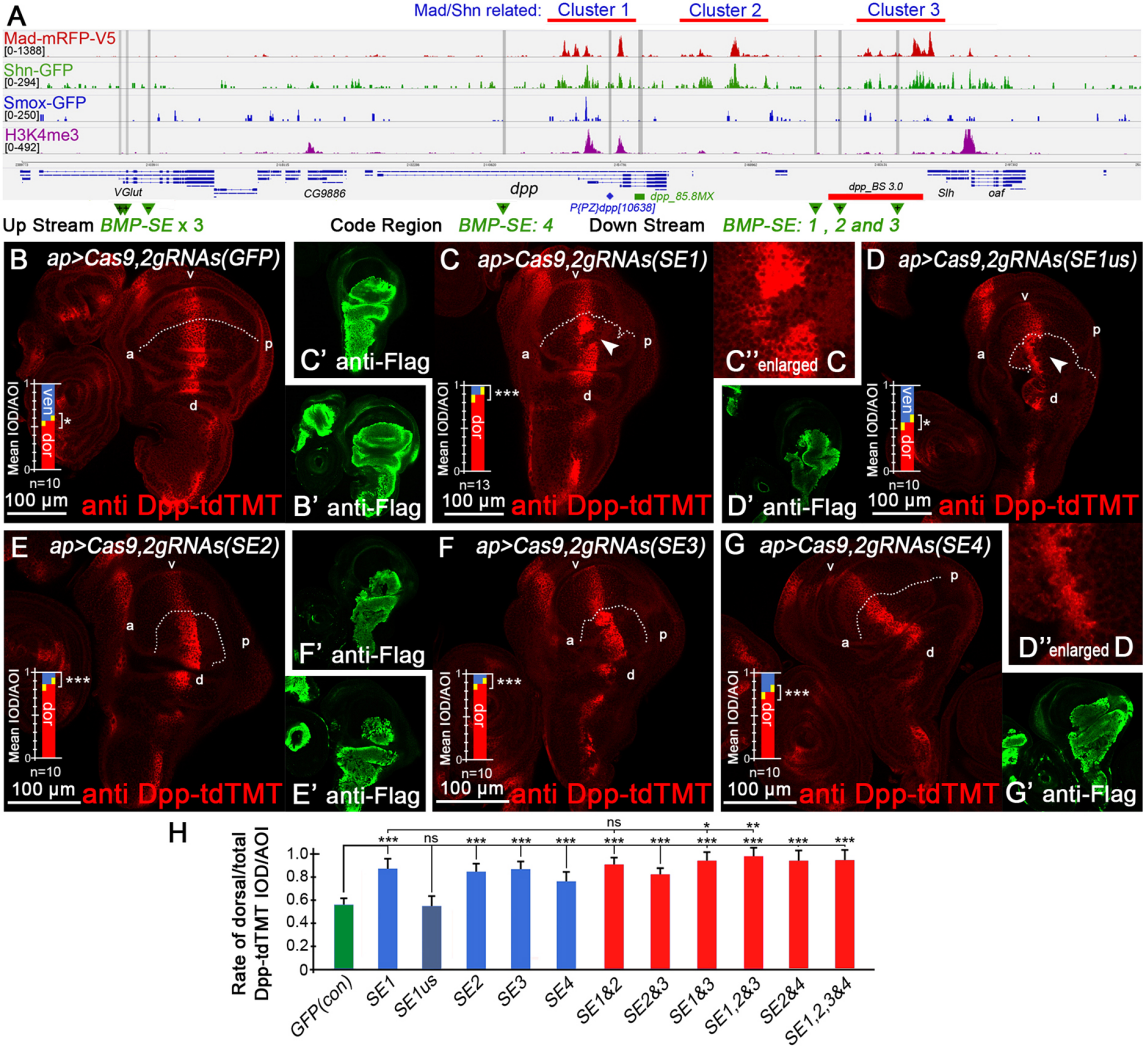
**Deletions of *BMP-SE* sites upregulate Dpp expression.** (A) Whole imaginal wing disc CUT&Tag peak map for Mad, Shn, Smox and H3K4me3 around the *dpp* gene locus, which highlights *BMP-SE* sites and regulatory regions mentioned in this study. (B-G) Local CRISPR/Cas9 deletion results of *BMP-SE* sites: (B) *gRNAs* for *GFP* as control, (C) *gRNAs* for *BMP-SE1*, (D) *gRNAs* for *BMP-SE1us*, (E) *gRNAs* for *BMP-SE2*, (F) *gRNAs* for *BMP-SE3* and (G) *gRNAs* against *BMP-SE4*. (B′-G′) Corresponding anti-Flag results. (D″) An enlarged view of D. (H) Collectively, these deletions elevated Dpp-tdTMT expression.

**Fig. 6.**
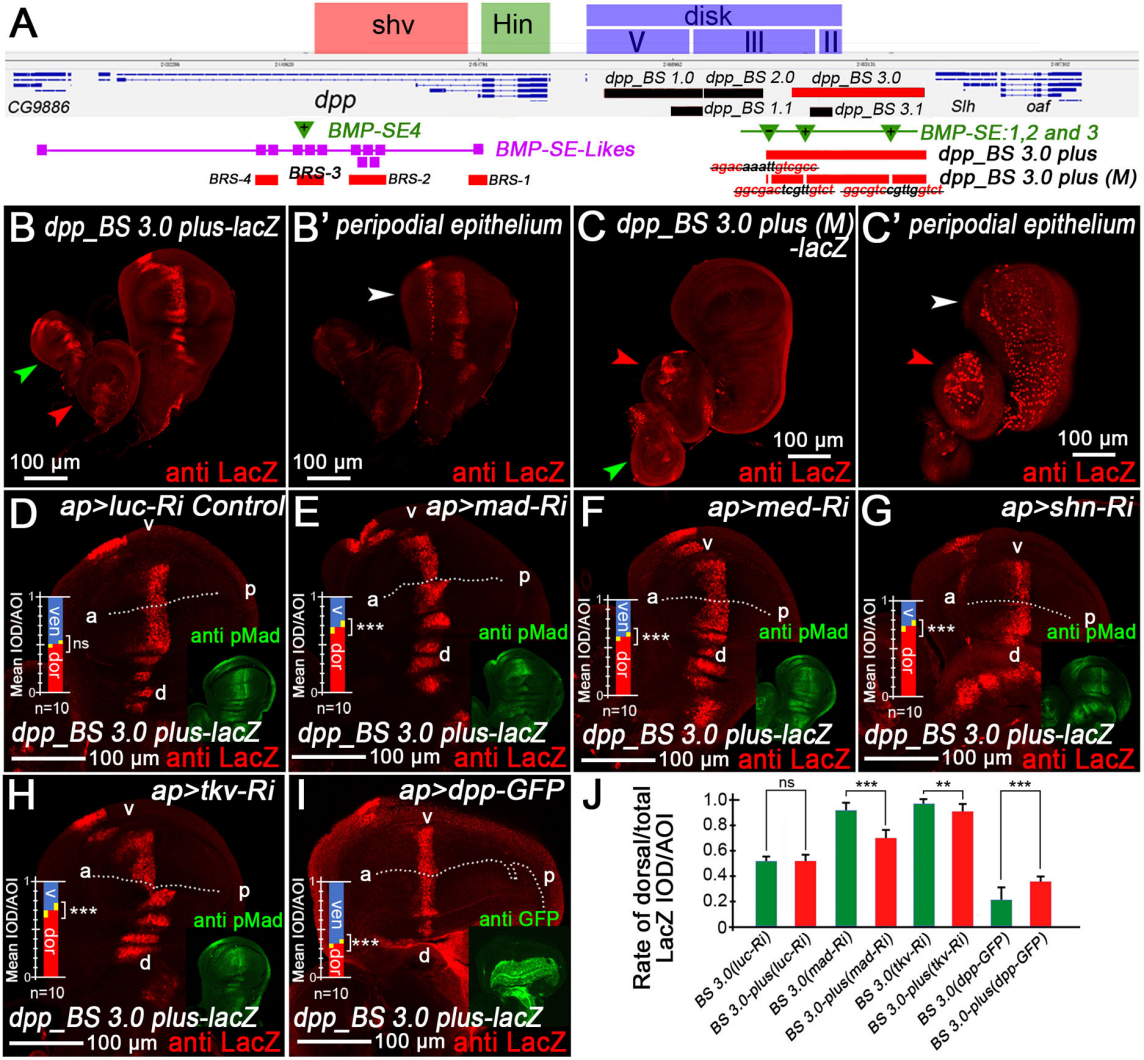
***BMP-SE* functional analysis through *in vivo dpp-lacZ* reporters.** (A) The *dpp* genomic map shows shv, Hin and disk regions, and associated regulatory fragments and motif sites. (B,B′) The staining of a newly developed *dpp_BS 3.0-plus-lacZ* reporter in the columnar epithelium layer (B) and peripodial epithelium staining (B′). (C,C′) Columnar epithelium layer staining of *dpp_BS3.0-plus (M)-lacZ* with three *BMP-SE* removed (C) and its LacZ staining in the peripodial epithelium (C′). White arrowheads indicate wing discs, green arrowheads indicate leg discs and red arrowheads indicate haltere discs. (D-G) RNA interference results of *dpp_BS 3.0-plus-lacZ* in (D) *ap>luc-RNAi* (control), (E) *ap>mad-RNAi*, (F) *ap>med-RNAi* and (G) *ap>shn-RNAi* groups shows upregulation of LacZ staining. (H,I) In the (H) *ap>dpp-GFP* and (I) *ap>dad-RNAi* groups, RNAi led to the downregulation of LacZ expression. (J) *dpp_BS 3.0-plus-lacZ* is less sensitive to the Dpp/BMP signaling pathway than *dpp_BS 3.0-lacZ*.

Having established that Dpp/BMP signaling represses *dpp* expression primarily through the BMP-SE complex, we investigated regulatory *BMP-SE* motifs around the *dpp* locus. We scanned 10 kb upstream to 30 kb downstream of *dpp*, identifying four *BMP-SE* sites (Fig. 5A): *BMP-SE1*, 2 kb upstream of *dpp-BS_3.0*; *BMP-SE2/3*, within *dpp-BS_3.0*; *BMP-SE4*, in the first intron of *dpp-RA*. Three additional upstream *BMP-SE* near *VGlut* were excluded based on earlier evidence that downstream disc region regulates *dpp* expression in imaginal discs (Masucci et al., 1990; Blackman et al., 1991). *BMP-SE4* (located in the shv region, Fig. 6A) played a minor role in regulating *dpp* in the larval wing disc. Therefore, detailed analyses of *BMP-SE4*, *BMP-SE-Like* elements in the shv region, and a luciferase assay identifying the responsive fragment *BMP-Response-Sequence-3* (*BRS-3*) (all locations shown in Fig. 6A) are presented in the Materials and Methods, with supporting data in Figs S10 and S11.

To validate *BMP-SE* function, we implemented a CRISPR/Cas9 system consisting of UAS-Cas9 and pU6-promoted ubiquitous *gRNAs*, each one targeting flanking sites at both sides. We generated fly strains targeting at single *BMP-SE* (*1*, 2, 3 and *4*) or double *BMP-SE* [(*1&3*) and (*2&4*)] sites. These strains were also recombined together to obtain flies with multiple deleted sites.

*gRNAs* targeting GFP served as controls and did not affect Dpp-tdTMT expression (Fig. 5B). When Cas9 targeted *BMP-SE1*, we observed significant upregulation of Dpp-tdTMT expression in the dorsal pouch (Fig. 5C). Concurrently, a high proportion (10/13) of Dpp-tdTMT-deficient clones (Fig. 5C″, white arrows) were detected in this group. To rule out off-target effects, we designed additional *gRNAs* (*SE1us*) to delete the upstream proximal region of *BMP-SE1*. We still observed a high proportion (5/10) of Dpp-tdTMT-deficient clones, but Dpp-tdTMT was not upregulated in the *SE1us* group (Fig. 5D). Dpp-tdTMT expression defects occurred less frequently at other sites (*SE2*, 0/10; *SE3*, 3/10; *SE4*, 0/10), suggesting that the region surrounding *SE1* may contain a critical enhancer for *dpp* transcription. Individual *gRNA*-mediated deletions at *SE2*, *SE3* and *SE4* also significantly upregulated Dpp-tdTMT expression in the pouch dorsal (Fig. 5E-G). These results indicate that all four *BMP-SE* sites regulate *dpp* expression, and disrupting any single site can activate *dpp* expression, particularly for *SE1, SE2* and *SE3* (Fig. 5H).

We subsequently combined *gRNAs* targeting downstream *BMP-SE* to generate *BMP*-*SE (1&2)*, *(2&3)*, *(1&3)* and *(1,2&3)* strains for multiple sites destruction. For *SE4* (located in the shv region), we created *SE (2&4)* and *SE (1,2,3&4)*. These results revealed high-frequency Dpp-tdTMT-deficient clones in all combined groups, likely due to large fragment deletions between sites, especially in *SE4-*containing combinations (Fig. S12F,G). Among these, the *SE (1,2,3&4)* group exhibited remarkable Dpp-tdTMT elevation with notum abnormalities and severe pouch deformation (Fig. S12G). The *SE (1,2&3) gRNAs* caused the most significant Dpp-tdTMT upregulation among all groups (Fig. S12E), accompanied by severe deformation but minimal notum effects. Finally, we excluded the Dpp-deficient clone regions from the images and quantitatively compared Dpp-tdTMT expression, finding that disrupting more *BMP-SE* likely induces greater upregulation of Dpp expression (Fig. 5H).

### Validation of *BMP-SE* function via *in vivo lacZ* reporters

Building on evidence that *BMP-SE* sites suppress Dpp expression, we provide direct transcriptional validation through *in vivo lacZ* reporter studies. We focused on *BMP-SE1*, *BMP-SE2* and *BMP-SE3* in the downstream disc region (Fig. 6A), which regulates larval-stage *dpp* expression and is subdivided into functional domains II, III and V based on phenotypic severity (Masucci et al., 1990; St Johnston et al., 1990).

As previously established, *dpp_BS 3.0-lacZ* (containing *BMP-SE2* and *3*) responds to Dpp/BMP signaling feedback (Fig. 3K-N). Since *SE1* lies outside this region but shows repressive function in CRISPR/Cas9 assays, we generated a *dpp_BS 3.0-plus-lacZ* reporter incorporating *SE1* (Fig. 6B,B′). This LacZ reporter was also repressed by the BMP-SE complex (Fig. 6E-G). Under normal conditions, both *dpp_BS 3.0-lacZ and dpp_BS 3.0-plus-lacz* showed similar expression, but diverged when signaling was modulated. In the *ap>tkv-RNAi*, *mad-RNAi*, *tkvQD* and *dpp-GFP* groups, *dpp_BS 3.0-lacZ* exhibited strong variation in the dorsal pouch (Fig. 3K-N). However, *dpp-BS3.0-plus-lacZ* maintained stability with minimal IOD/AOI fluctuation (Fig. 6H,I), indicating that the *SE1* site enhances regulatory robustness. The two *lacZ* strains comparison chart is show in Fig. 6J. *dpp_BS 3.0-plus-lacZ* shows less sensitivity – demonstrating that additional *BMP-SE* sites enhance repression efficiency and signaling tolerance.

Deletion of all three *BMP-SE* motifs *GRCGNC(N)_5_GTCT* from *dpp_BS 3.0-plus-lacZ* abolished disc proper layer expression (Fig. 6C), but expanded and expression enhanced in peripodial epithelium and haltere disc (Fig. 6C′). This demonstrates *BMP-SE* also contribute to basal *lacZ* reporter transcription under specific conditions in distinct tissues, which may indirectly affected activators or disrupted the CRM (cis-regulatory module) architecture. CRISPR-mediated disruption of *SE2/3* in *dpp_BS 3.0* and *SE1/2/3* in *dpp_BS 3.0-plus* induced expected upregulation (Fig. S13A-E). These results demonstrated that the *dpp_BS 3.0-plus* region functions as a dual-function module: both an enhancer and a *BMP-SE-*dependent repressor of transcription.

### Varying Dpp/BMP signaling alters Mad-DNA interactions

**Fig. 7.**
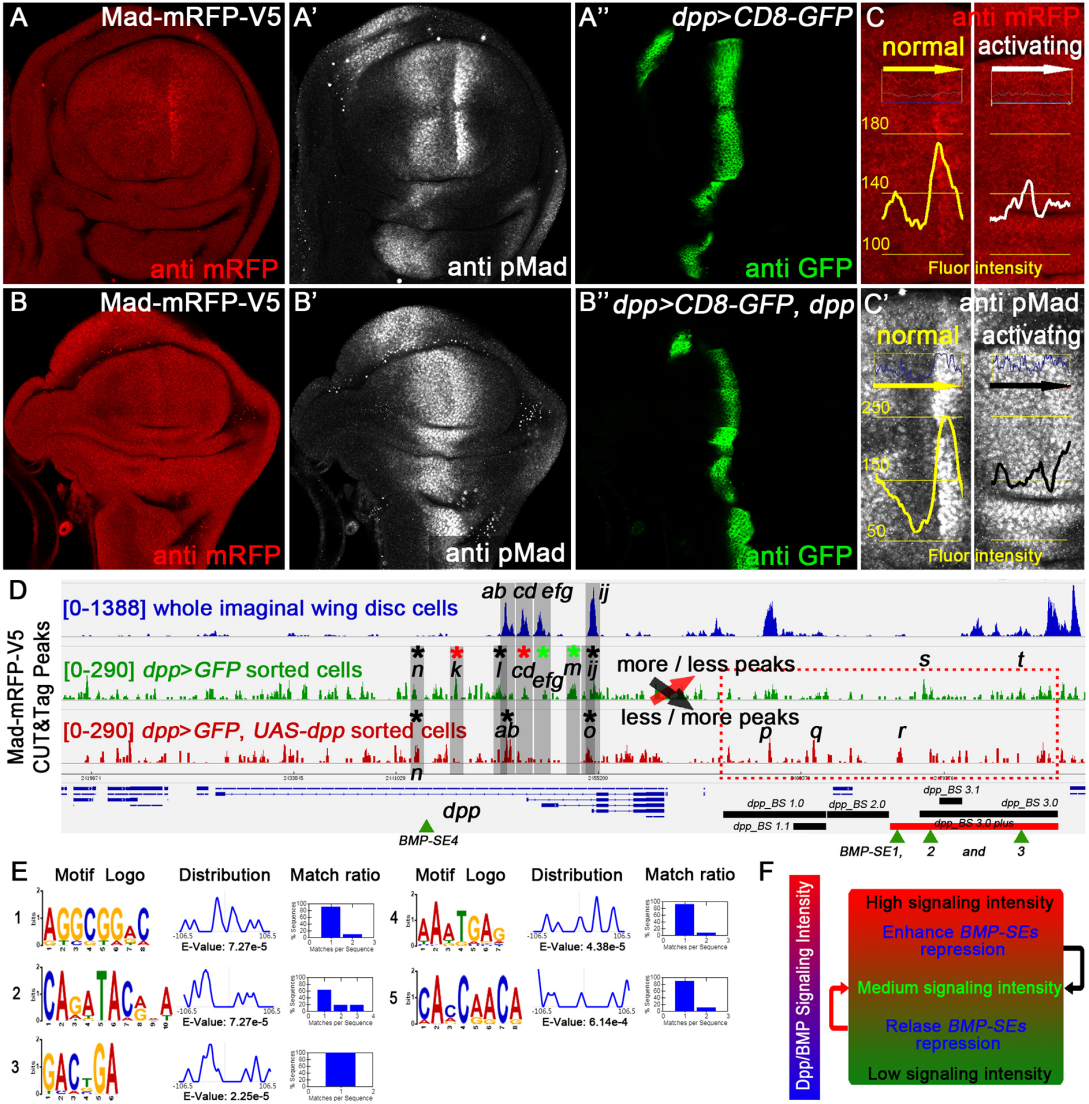
**Discovery of Mad-DNA interaction and previously unreported motifs via CUT&Tag assay.** (A and C left) Mad-mRFP-V5 staining, (A′ and C′ left) pMad staining and (A″) GFP staining in *dpp>CD8-GFP* in a normal group wing imaginal disc. (B and C right) Mad-mRFP-V5 staining, (B′ and C′ right) pMad staining and (B″) GFP staining in a *dpp>CD8-GFP, dpp>dpp* activating group. (C,C′) Statistics of fluorescence intensity through antibody staining, demonstrating that Mad protein is also negatively regulates by BMP signaling in the *dv*-axis adjacent to Dpp-producing cells. (D) CUT&Tag peaks of Mad-mRFP-V5, where the upper blue peaks represent whole imaginal disc peaks, the middle green peaks represent *dpp>CD8-GFP* sorted cells (normal group), and the bottom red peaks represent *dpp>CD8-GFP* and *dpp* sorted cells (activating group). The lowercase letters *a* to *t* represent the different peaks we identified [where *n* is the control fragment (in which the two groups have similar peaks)]. The regulatory role of each fragment is indicated by black asterisks (no difference), red asterisks (upregulation) and green asterisks (downregulation). (E) Previously unreported Mad-associated DNA binding motifs identified in this study. (F) A general model of Dpp/BMP signaling via the BMP-SE complex that modulates signaling homeostasis.

We performed CUT&Tag assays for Mad, Shn, Smox and H3K4me3 in wing discs to assess BMP-SE-related binding (Fig. 5A). Due to the lack of effective antibodies against Mad, we generated an endogenous mad-mRFP-V5 tagging strain (Fig. 7A-C′), with tandem mRFP-V5 tags inserted into the middle of Mad-PB or the N-terminus of Mad-PA. Additional CUT&Tag assays were performed using anti-GFP antibodies for *Shn-GFP* and *Smox-GFP*, and anti-H3K4me3 for *W^1118^* strain.

Initially whole-disc CUT&Tag (Fig. 5A and Fig. S9B) revealed active chromatin around *dpp-RC* and *RE*, consistent with RT-qPCR data (Fig. S9A,A′). Mad peaks partially overlapped Shn peaks (Fig. 5A and Fig. S9B), suggesting BMP-SE complex involvement. The results of multiple sequence motif analyses using XSTREME at MEME suite (Bailey et al., 2015; Bailey, 2021) are shown in Fig. S7E; and the single-fragment motif identification via STAMP data (Mahony and Benos, 2007) are shown in Fig. S9C. This information revealed transcription factor binding sites, including Mad, Med, Brk and others, indicating also existing regulatory mechanisms beyond the *BMP-SE* modules. We subdivided Mad-mRFP-V5 clusters 1-4 into segments *ab*, *cd*, *efg*, *h* and *ij*, with low-abundance peak *h* containing a ventral vein-deficient (Vvl) motif (de Celis et al., 1995) (Fig. S9C).

Consistent with pMad staining (Fig. 7A-C′), the most Mad peaks were detected in Dpp-receiving rather than Dpp-producing cells. To examine Dpp-producing cells specifically, we performed CUT&Tag assays from cell sorting: a control group (*dpp>GFP*; Fig. 7A-A″ and left panels in Fig. 7C,C′) and a BMP-activated group (*dpp>GFP and dpp;* Fig. 7B-B″ and right panels in Fig. 7C,C′).

These assays revealed distinct Mad-mRFP-V5 peak patterns: controls showed more peaks in the shv region but fewer in the disc region, while BMP-activated samples exhibited the inverse pattern. The peak *s* overlapped *BMP-SE2*, and peak *t* neighbored *BMP-SE3*. Under normal signaling, *BMP-SE2* was the dominant binding site with secondary *BMP-SE3* binding. Enhanced signaling strengthened peak *r* (coincides with *BMP-SE1*), while *BMP-SE1* and *SE2* became dominant, and *SE3* binding diminished. According to chromatin looping models (Li et al., 2006; Kim et al., 2018; Perillo et al., 2024), such dynamic masking may prevent Smad complex binding at *dpp* TSSs, inhibiting transcription by total Mad peaks reduction in the shv-region. MEME motif analysis identified multiple motifs beyond classical *BMP-SE* sites (Fig. 7E), with the top motif AGGCGGAC potentially serving as a Mad binding site due to similarity to GNCGRC in *BMP-SE* and GGCGCCA in *BMP-AE*. Variable bases (*N*/*R*) and 5-bp gaps in classical *BMP-SE* motifs likely confer differential Smad complex affinity. Thus, varying signaling strengths favor distinct binding that recruit different co-factors for gene regulation. We propose a global regulatory model integrating these findings (Fig. 7F). The *dpp*-isoform identification primers and core sequences of peaks a to t for XSTREME and STAMP analysis are described in the supplementary Materials and Methods.

### Additional experimental content – functional analysis of *BMP-SE4* containing a *BRS-3* segment in the shv region

Another *BMP-SE* regulatory site, *SE4*, is located in the middle of the first intron of *dpp-RA*; we have previously conducted studies on feedback regulation in the *dpp* shv region. In addition to *BMP-SE4*, fragment *BRS-3* contains many *BMP-SE-like* sites. When defining *BMP-SE-like* sequences, we analyzed motifs containing a typical Med-binding site (*GTCT*) and allowed up to two base mismatches in the Mad-binding sequence (*GRCGNC*). Notably, three *BMP-SE-like* sites are located near *SE4*, suggesting the presence of a potential regulatory cluster. Using this approach, we identified four *BMP-responsive* segments (*dpp-BRS-1* to *dpp-BRS-4*) (Fig. 6A). *BMP-SE* and three *BMP-SE-like* motifs, with detailed information provided in the supplementary Materials and Methods. Through *in vitro* luciferase assays, we found that the inhibitory ability of *dpp-BRS-1*, *2* and *4* segments is limited, but *dpp-BRS-3* displayed some repressive activity (Fig. S10A). The genomic sequences of *dpp-BRS-1* to *4* and their *BMP-SE(-Like)* motif information are described in the supplementary Materials and Methods.

We then cloned the *dpp-BRS-3* fragment into a *lacZ* enhancer reporter construct. For enhanced visibility, we repeated this sequence three times, and all data presented in this work were obtained from triplicate reporter strains, which provide better LacZ staining than monomers. Results implied that, in the Dpp-producing region, *dpp-BRS-3-lac*Z showed weak expression in the dorsal part as scattered dots (Figs S10B, S11A,C). Outside the Dpp-producing cells, *dpp-BRS-3-lacZ* showed a strong response to Dpp/BMP signaling in proximal Dpp-receiving cells, as indicated by the four stripes in Figs S10B,C′ and S11A,C. In the Dpp-producing region, its expression is very limited. The main functional area corresponds to the developmental positions of longitudinal wing veins L3 and L4. During the larval stage, this segment seems not to play a major regulatory role but may regulates *dpp* expression in wing vein during the pupal stage.

In parallel, we generated a negative control strain as *dpp-BRS-3-control-lacZ* by removing all the *BMP-SE* and three *BMP-SE-like* sites from the original genomic sequence (Figs S10D and S11B). This *dpp-BRS-3* control strain almost completely lost all detectable LacZ staining (Figs S10D, S11B). These results reveal that the basal expression of *dpp-BRS-3-lacZ* primarily depends on Dpp/BMP signaling. Additionally, *dpp-BRS-3-lacZ* can serve as a reporter in Dpp-receiving cells, including the cells adjacent to the four stripes and those in the air sac (Figs S10C′, S11A). However, *dpp-BRS-3-lacZ* was nearly undetectable in the Dpp-producing region, with only faint staining observed in the dorsal pouch (pattern shown in Figs S10C′ and S11C). In addition, we knocked out *BMP-SE4* while retaining the three *BMP-SE-like* sites, and the basic properties of *BMP-SE-like* (M) were not significantly different from original *dpp-BRS-3* (Fig. S10L-N). This suggested that multiple *BMP-SE-likes* function to create a positive response to BMP signaling. Removing *BMP-SE* fragment led to slightly released LacZ expression form restriction (Fig. S10H,N).

The expression of both *dpp-BRS-3* and *dpp-BRS-3 (M)* depend on Dpp/BMP signaling. Four L3 and L4 stripes are positively response to activation of signaling, and LacZ expression is sharply reduced upon knockdown in *tkv* or *mad-RNAi* group (Fig. S10E,F). However, overexpression of TkvQD or Dpp-GFP could reduce but not eliminate the stripes, and stably induces a large strip that is elongated with *ap*-axis in the posterior-dorsal region (Fig. S10G,H,N). The reduction of the L3 and L4 stripes in ventral region may result from an overall Dpp concentration decrease, led by Dpp dynamical diffusion in pouch. Fig. S10J,K shows that knockdown of *med* and *shn* also increases *dpp-BRS-3-LacZ* staining in the dorsal region. The base pairs of *BMP-SE* sites are hypothesized to have flexibility, which may allow them to adapt to bi-directional regulation, functioning as either inhibitory or activating, depending on the local signaling strength.

We further investigated *dpp* TSS-upstream fragments using qPCR-based *in vitro* reporter assays, here focusing on shv and Hin regions, which exhibited denser peaks in controls (Fig. S14). Cloned regulatory fragments were inserted between the 2.4 kb *wg-*promoter and *Hsp70*-promoter of the *lacZ* reporter construct. In transfected S2 cells, fragments *cd* and *k* responded positively to Dpp/BMP signaling, while *efg* and *m* responded negatively. The primers for constructs and qPCR are listed in the supplementary Materials and Methods.

To exclude indirect effects from the known *dpp* regulators Engrailed (Shen and Dahmann, 2005), Hedgehog (Hepker et al., 1999) and Wnt (Sotillos and de Celis, 2006), we examined key pathway components and found no alterations in Engrailed (En), Distal-less (Dll), Patch (Ptc) and Cubitus interruptus (Ci) expression during Dpp/BMP-*dpp* feedback assays (Fig. S15A-G‴).

This study establishes that *Drosophila* Dpp/BMP signaling represses its own expression through BMP-SE complex-mediated transcriptional silencing, with this negative feedback maintaining signaling homeostasis by stabilizing ligand dosage.

## DISCUSSION

### Source-sink theory and Dpp autoregulation

The source-sink theory explains morphogen gradient patterning through balanced ligand production (source) and distributed removal (sink), ensuring developmental precision. Our study extends the theory by revealing that a transcriptional feedback mechanism autoregulates Dpp production during wing imaginal disc development in *Drosophila*. This feedback loop aligns with the robustness theory proposed by Eldar et al. (2002, 2004), which suggests that Dpp/BMP signaling maintains stability through multiple regulatory layers. The findings indicate that the Mad/Med/Shn complex, particularly through *BMP-SE* motifs, plays a central role in this robustness by fine-tuning *dpp* expression in response to signaling intensity. This mechanism not only stabilizes Dpp gradients but also buffers against fluctuations in ligand production, ensuring precise tissue patterning and development.

### Sensitivity of Dpp-producing cells to signaling intensity

The study reveals that Dpp-producing cells are high sensitivity to extracellular Dpp ligand concentration, which feeds back to regulate *dpp* transcription. This sensitivity is mediated by low-level expression of the type I receptor Tkv in the Dpp-producing region (Figs S10B′ and S11C′). This finding challenges the traditional view that Dpp/BMP signaling is primarily required in receiving cells, emphasizing its crucial role in monitoring ligand dosage within producing cells. In contrast, the type I receptor Sax and type II receptor Punt show ubiquitous expression in the wing disc (Fig. S6A,B), indicating that restricted Tkv expression prevents signaling overactivity and establishes optimal Dpp gradients.

### Dose compensation and allelic interaction

The *dpp* gene exhibits dose-dependent lethality, reflecting its crucial role in development. Our results demonstrate that *dpp* alleles partially compensate for the loss of one another through transcriptional upregulation, although this compensation is insufficient to fully rescue pMad levels (Fig. 1E′ and Fig. S5A‴) or completely restore Sal expression over a long range (Fig. S5B‴,C″). This dose compensation mechanism highlights the robustness of the pathway, allowing gene dosage flexibility while maintaining functional output. However, the inability of single alleles to fully meet physiological requirement reveals the limits of this regulatory mechanism.

### Discrepancy between Cut&Tag peaks and predicted motifs

The observed mismatch between Cut&Tag peaks and predicted motif locations stems from multiple factors. Regulatory proteins frequently bind DNA indirectly through interactions with transcription factor or chromatin remodelers rather than direct motif recognition. Chromatin architecture (particularly looping) enables interactions with distal regions lacking canonical motifs. Technical considerations include Tn5 transposase preference for accessible chromatin and potential antibody disruption by protein conformation. Additionally, low-affinity binding sites often fail to generate strong peaks. These mechanisms collectively explain the partial coincidence between observed protein-DNA binding peaks and predicted motifs.

### Conclusion and future directions

This study elucidates a transcriptional feedback mechanism ensuring precise Dpp control in the *Drosophila* wing disc. Mediated by Mad/Med/Shn and *BMP-SE* motifs, this system demonstrates developmental signaling robustness. Future research should address: (1) the molecular coordination between Mad/Med/Shn and repressors; (2) the chromatin remodeling in BMP-SE complex regulation; and (3) the conservation of these regulatory principles in other developmental contexts. Investigating these fields will advance understanding of Dpp/BMP signaling in development and disease.

## MATERIALS AND METHODS

### Fly stocks

The following fly stocks were generated in our laboratory specifically for this study: *dpp-sfGFP*, *dpp-tdTMT*, *mad-mRFP-V5*, *tkv-sfGFP*, *sax-mRFP-V5, punt-YFP (Venus), dpp-BRS-3-lacZ, dpp-BRS-3-control-lacZ* and *dpp-BRS-3 (M)-lacZ, dpp_BS 3.0-plus-lacZ, dpp_BS 3.0-plus (M)-lacZ*, and the CRISPR/Cas9 related strains *UAS-Flag-Cas9-dU6-dual-gRNAs* [(*BMP-SE1*), (*BMP-SE1us*), (*BMP-SE2*), (*BMP-SE3*), (*BMP-SE4)* and (*GFP*) as control] and *UAS-Flag-Cas9-dU6-quadra-gRNAs* [(*BMP-SE1&3*), (*BMP-SE2&4*)]. *UAS-dpp-GFP* and *dpp-Gal4* were gifts from Stephen Cohen (European Molecular Biology Laboratory, Heidelberg, Germany). *ap-Gal4* (BL3041), *mirr-Gal4* (BL29650), *tub-Gal80^ts^* in 2nd chromosome (BL7019), *Tub-Gal80^ts^* in 3rd chromosome (BL7017), *UAS-flippase* (BL8209), *UAS-GFP-RNAi* (BL41553), *UAS-luc-RNAi* (BL35788), *UAS-mad-RNAi* (BL31316), *UAS-tkv-RNAi* (BL31316), *UAS-Shn-RNAi* (BL34689), *UAS-med-RNAi* (BL31928), *UAS-dad-RNAi* (BL33759), *UAS-brk-RNAi* (BL51789), *UAS-tkvQD* (BL36536), *dpp-LacZ[10638]* (BL12379), *dpp-LacZ [BS 3.0]* (BL5527), *UAS-dpp* (BL1486), *Shn-GFP* (BL42671) and *Smox-GFP* (BL43958) are from the Bloomington Drosophila Stock Center. *dpp[S4]* (DGRC:106645) and *dpp[H46]* (DGRC:108956) are from KYOTO Drosophila Stock Center.

### Fly breeds and genotypes

All *tub-Gal80^ts^* carrying crosses were kept at 25°C until they reached 2nd instar larvae, when they were transferred to 29°C for final staining or cell sorting. Exceptionally, *ap>tkvQD*, *tub-Gal80^ts^* strains are partially lethal at 25°C, so they were kept at 20°C and then 2nd instar larvae were transferred to 29°C until dissection. While most strains were worked well at 25°C, *med-RNAi* exhibited improved effectiveness at 29°C. Detailed genotypes for all the figures are provided in Table S1.

### Antibodies and immunofluorescence staining

All imaginal wing and eye discs were dissected from *Drosophila* third instar larvae. They were fixed with 4% fresh paraformaldehyde (PFA, Sigma, V900894) solution for 20 min at room temperature, then washed three times for 5 min each. The blocking and antibody incubation buffers were PBST (0.1% Triton X-100) with 5% fetal bovine serum. Primary antibodies used were as follows: chicken anti-GFP (1:1000, Abcam, ab13970), rabbit anti-GFP (1:400, Invitrogen, A-11122), rabbit anti-pMad (1:400, CST, 41D10), rabbit anti-RFP/tdTMT (1:1000, Rockland Immunochemicals, 600-401-379), rat anti-Sal (1:400, lab-made), chicken anti-β-Galactosidase (1:1000, Abcam, ab134435), mouse anti-Ptc (1:50, DSHB, Apa1), rat anti-Ci (1:50, DSHB, 2A1), mouse anti-Engrailed (1:100, lab-made), goat anti-Distal-less (dF-20, 1:100, Santa Cruz, sc-15858) and mouse anti Flag [1: 1000, Sigma, F3165 (clone M2)].

Fluorescence-conjugated secondary antibodies were from Jackson ImmunoResearch Laboratories and were used at 1:400 dilution: donkey anti-chicken Alexa Fluor 488 (A11039), donkey anti-rabbit Alexa Fluor 488 (A21206), donkey anti-rabbit Alexa Fluor 546 (A10040), donkey anti-rat Alexa Fluor 647 (A48272), goat anti-chicken Alexa Fluor 546 (A11040) and donkey anti-mouse Alexa Fluor 488 (A21202). Other reagents used were DAPI (4′,6-diamidino-2-phenylindole,dihydrochloride, Sigma,D9542) and SlowFade Gold Antifade Mountant (Invitrogen, S36937).

### *In situ* hybridization

The wing disc *in situ* hybridization for the detection of *dpp* mRNA follows the protocol described by Toledano et al. (2012). Larvae were dissected in ice-cold PBS, then fixed in pre-chilled 4% fresh PFA solution for 30 min in room temperature. Samples were washed with ice-cold PBST (0.1% Triton X-100) three times: twice for 5 min each, then for 20 min on ice. Pre-hybridization was carried out in buffer [5×SSC; 50% (v/v) formamide; 20% 5×blocking reagent; 0.1% (v/v) Tween 20; 50 μg/ml heparin; 50 μg/ml yeast tRNA] for 1 h at 55°C. The buffer was replaced with fresh pre-hybridization buffer containing 100 ng/ml RNA probe, mixed well and incubated at 55°C for at least 6 h. Samples were washed with hybridization base buffer solution [5×SSC; 50% (v/v) formamide; Tween-20, 0.1% (v/v)] twice at 60°C for 1 h each, then washed with PBST three times for 5 min each. Samples were blocked using 5% fetal bovine serum in PBST solution for 30 min, then incubated with antibodies (goat anti-Digoxigenin-POD, Roche, 1:200; chicken anti-GFP, 1:1000; rabbit anti-TdTMT, Rockland Immunchemicals, 1:1000) for at least 4 h at 4°C. Samples were then washed with PBST three times for 5 min each and incubated in fluorescence-conjugated Alexa Fluor second antibody (at 1:400) for 1 h at room temperature. TSA regent was diluted in kit buffer (1: 100) and samples incubated for 20 min at room temperature to develop the staining. Samples were washed with PBST three times for 5 min each, then mounted with anti-fade reagent for confocal observation.

Other *in situ* hybridization reagents include: T7 RNA polymerase (NEB, M0251L), Blocking Reagent (Roche, 11096176001), Dig-RNA Labeling Mix (Roche, 11277073910), anti-Digoxigenin-POD, Fab fragments (Roche, 11207733910), TSA Plus Fluorescein Systems for *in situ* hybridization (PerkinElmer), TSA-TRITC(SAT702001KT) and TSA-Cyanine (SAT705A001KT). The primer sequences for mRNA probe synthesis are outlined in the supplementary Materials and Methods.

### Tissue and cell culture

*Drosophila* S2 cells were cultured at 25°C in Schneider's *Drosophila* Medium (Gibco, 21720024) with 5% fetal bovine serum (Gibco, 10100147). All transfection experiments were carried out using Effectene Transfection Reagent (Vazyme, T101). 24-well plates were used for *lacZ* reporter assays (Fig. S14). Each well was seeded with 5×10^6^ S2 cells, allowing them to grow to 70-80% confluency, then transfected with 100 ng *lacZ* reporter plasmid. In the BMP activation groups, an additional 30 ng *pAC-TkvQD* plasmid was added. Cells were harvested after 48 h and used for RT-qPCR. Shields and Sang M3 insect medium (Sigma, S8398) was used for extracellular staining (Fig. 1C′,D).

### RT-qPCR

Total RNA was extracted from S2 cells or wing, eye-antenna and leg imaginal discs from 20 third instar larvae using a FastPure Cell/Tissue Total RNA Isolation Kit (Vazyme, RC101). Complementary DNA (cDNA) was prepared using HiScript II Q Select RT SuperMix for qPCR (+gDNA wiper) (Vazyme, R233). qPCR was performed using AceQ Universal SYBR qPCR Master Mix (Vazyme,Q511) on a BioRad CFX96 Touch system. Experiments were carried out with three independent biological replicates, each containing three technical replicates in qPCR. The ribosomal gene *RpL32 (rp49*) was used as an internal reference. Primers for qPCR are listed in the supplementary Materials and Methods.

### Luciferase assay

Approximately 5×10^6^ S2 cells were seeded into each well of 24-well plates. When cells reached 70-80% confluency, each well was transfected with 5 ng *Renilla-luciferase*, 30 ng *dpp-BRS-X* reporter and 30 ng *pAC-TkvQD* plasmids. After 48 h, S2 cells were washed with PBS and then lysed with buffer from the assay kit. *Renilla-luciferase* and *Firefly-luciferase* activity were detected using a Dual *Luciferase* Reporter Assay Kit (Vazyme, DL101) with a Promega GloMax 96 as the scanning equipment. The results represent three independent experiments, each with three technical replicates. The primer sequences of constructs for the *luciferase* assay are listed in the supplementary Materials and Methods.

### CUT&Tag assay

Trypsinization and reduction of the tissue to a unicellular state of third-instar larvae wing imaginal disc with Trypsin-EDTA solution (0.05% Trypsin, beyotime, C0202) at 37°C for 2 min, followed by pipetting at room temperature. After serum termination of digestion and cleaning cells with PBS solution, Flow cytometry (BD FACSAria II) sorting was adapted to Dpp-producing GFP^+^ cells. For whole discs, samples were directly adhered to ConA beads (include in kit).The detailed protocol for the CUT&Tag assay follows the manufacturer's instructions in the assay kit Hyperactive *In-Situ* ChIP Library Prep Kit for Illumina (pA-Tn5) (Vazyme,TD902). Antibodies used were: rabbit anti-GFP (1:400, Invitrogen, A-11122), rabbit anti-RFP/tdTMT (1:400, Rockland Immunochemicals, 600-401-379), rabbit anti-V5 (1:400, Abcam, ab9116) and rabbit anti-H3K4me3 (1:400, CST, 9751). TruePrep Index Kit V4 for Illumina (Vazyme, TD204) was used for DNA segment amplification. VAHTS DNA Clean Beads (Vazyme, N411) were used for DNA purification. The high-throughput sequencing platform was Illumina Novaseq 6000. Analysis and visualization software included Deseq and IVG 2.0. Raw sequencing data have been deposited in the NCBI SRA database under accession numbers SRR18532285, SRR18532289 and SRR15734945.

### Quantification and statistical analysis

Fluorescence intensity analysis was performed using ZEN 2.0 imaging software (Carl Zeiss). For quantitative analysis of area, regions of interest (AOIs) were defined by the entire pouch or dorsal region. For pMad, Sal, LacZ, *in situ* signal and fluor-Dpp, et., the AOI defined by actually expressed region. The fluorescence intensity was converted to integrated optical density (IOD) measurements within the area, regions of interest (AOI), which were conducted using the Image-Pro Plus software (Version 6.0, Media Cybernetics). The IOD values were normalized to the area of the to account for variations in fluorescence intensity, and IOD alone to calculate total expression level. Due to the variation in expression between samples, we conducted a ratio analysis using the ratios of dorsal and ventral (or entire pouch) comparisons, and then combined the ratios of each sample for statistical analysis. If there is no internal comparison, we will increase the sample size for statistical analysis, e.g. the embryo statistics. The statistics related to width measuring in the images still used Image-Pro Plus 6.0 software. The significance of paired comparison samples was detected using a *t*-test. Error bars indicate the standard deviation; results are considered to be statistically significant as follows: *P<0.05, **P<0.01 and ***P<0.001.

## Supplementary Material

10.1242/develop.204488_sup1 Supplementary information
