# Supplementary material for: Drosophila Dpp and BMP signaling directly regulates dpp transcription for optimal ligand production
Source: Development. 2025 Nov 10;152(21):dev204488. doi: 10.1242/dev.204488 (PMC12633792; doi:10.1242/dev.204488)
Supplement: Supplementary information [file develop-152-204488-s1.pdf]

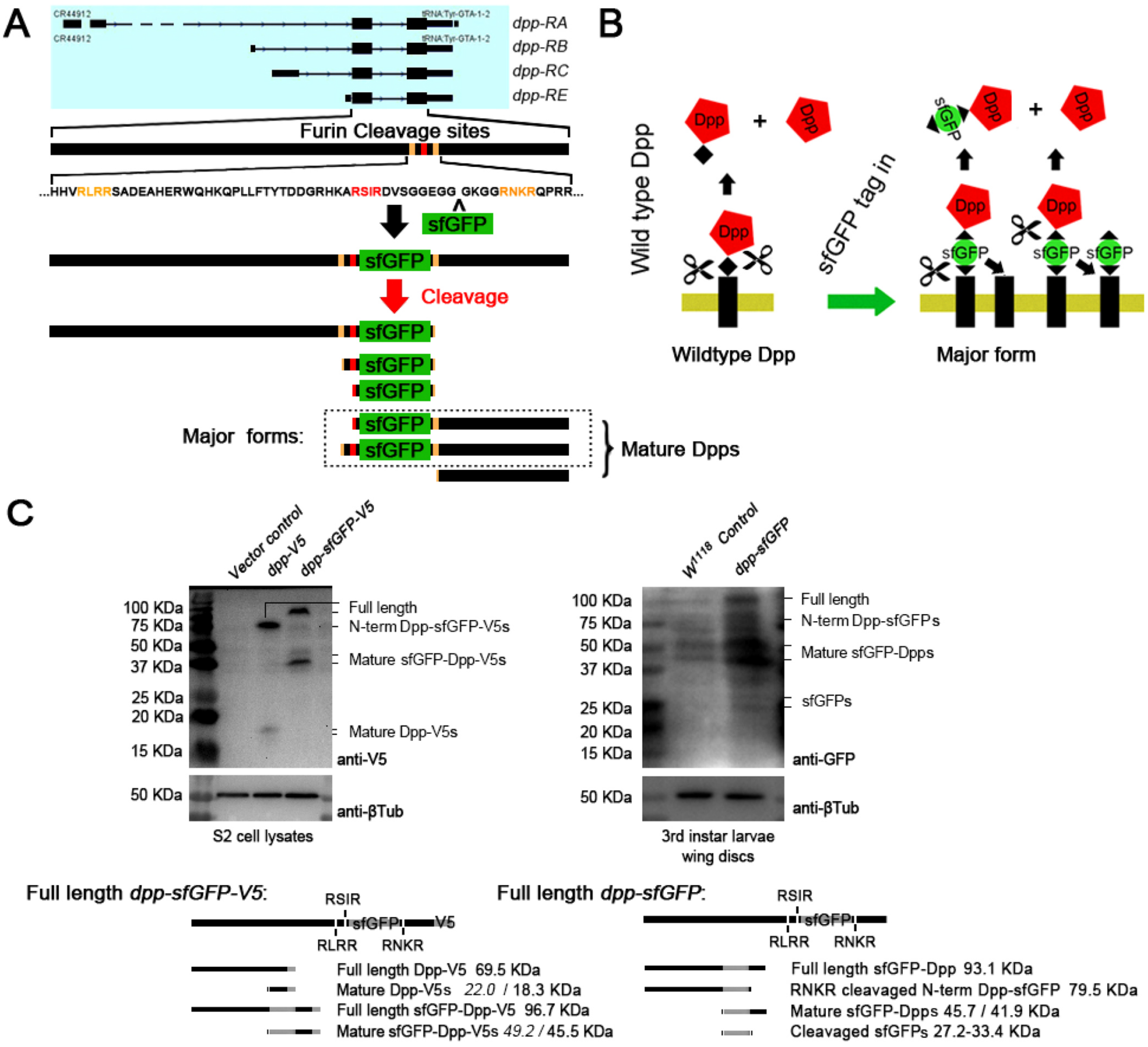

Fig. S1. Molecular properties of Dpp-sfGFP prepared in the study.

(A) Schematic of the sfGFP insertion site and predicted molecular cleavage sites. (B) GFP fluorescence primarily labels immature pro-Dpp, mature Dpp, and intracellular retained forms. (C) Western blot analysis of the Dpp-sfGFP cleavage in *in vitro* S2 cells and 3rd instar larval wing imaginal discs.

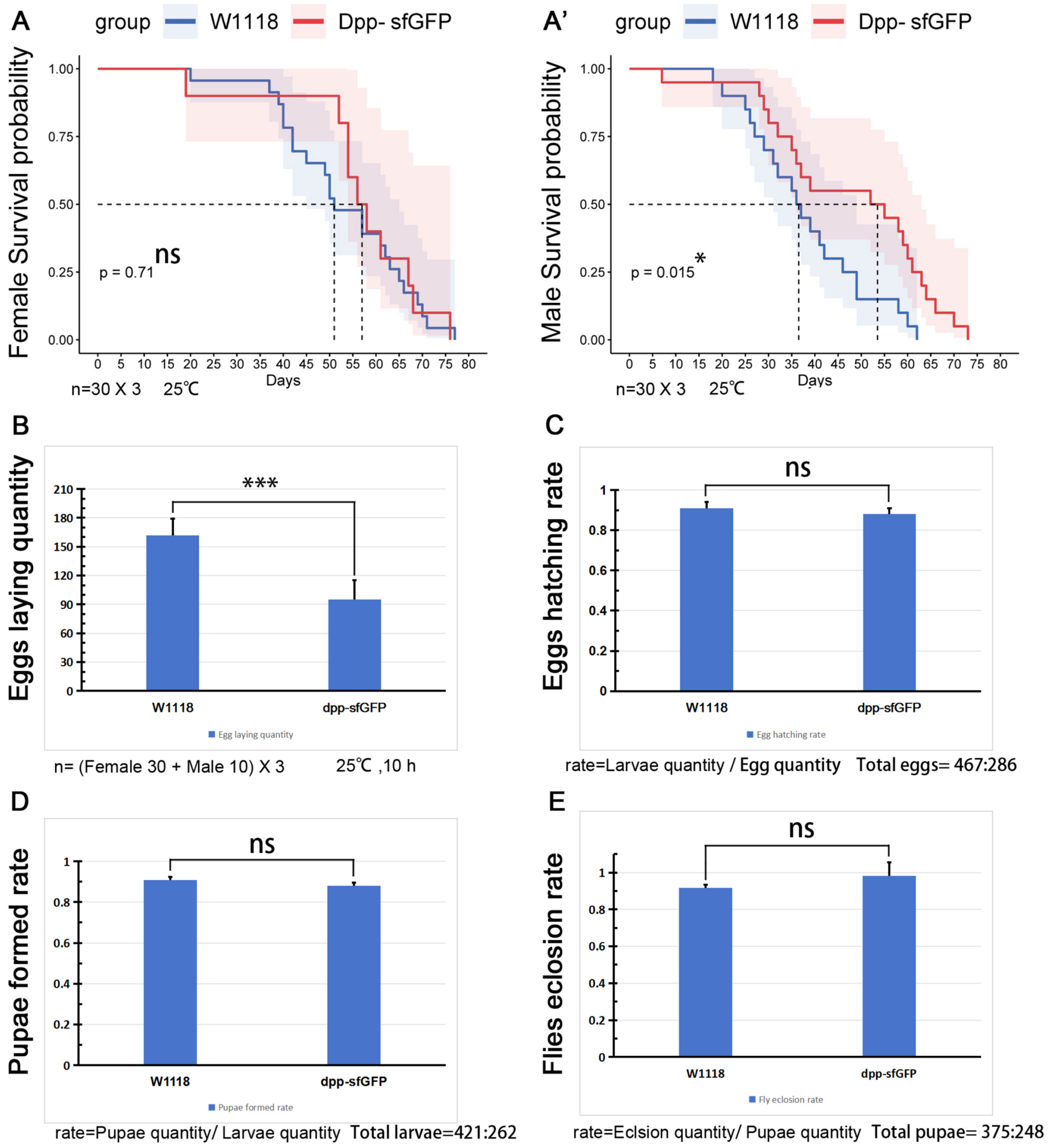

Fig. S2. Analysis of survival curves and the rates of spawning, hatching, pupation, and eclosion.

(A) While a small number of early deaths occurred initially, overall survival of *dpp-sfGFP* female flies did not significantly differ from controls (W^{1118}). However, between 15-55 days of age, their survival rate was slightly better than that of the control group, and the survival curves overlapped in later stages. (B) In male adult flies, *dpp-sfGFP* flies exhibited better survival performance, with statistically significant results ($p<0.05$), and the median mortality was delayed by nearly 20 days. (C) Female flies exhibited significantly reduced fecundity (~40% fewer eggs than W^{1118}). However, no significant differences were observed in hatching rate (D), pupation rate (E), or eclosion rate compared to the control group.

Development • Supplementary information

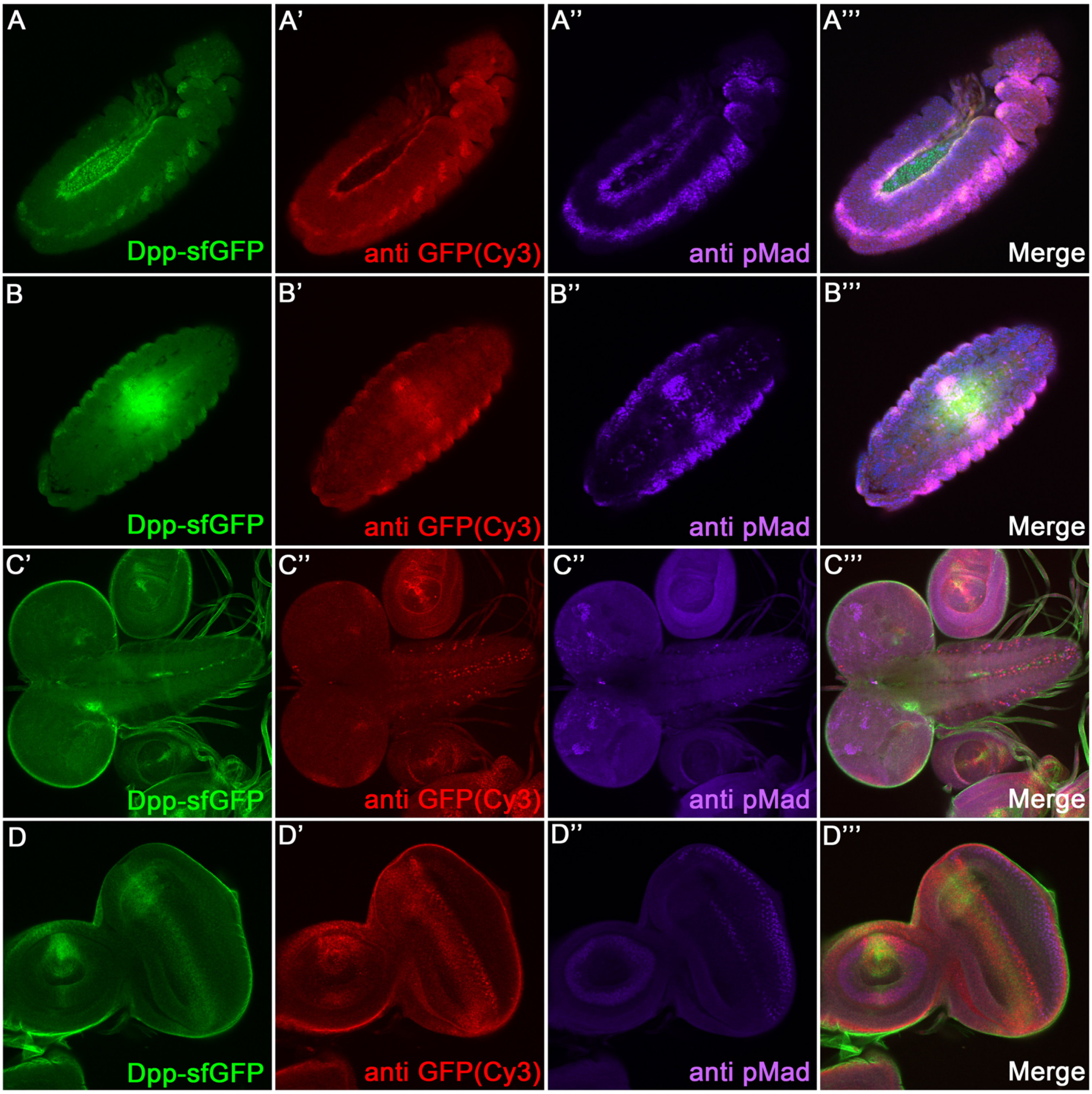

Fig. S3. Expression and distribution of Dpp-sfGFP in different *Drosophila* tissues and embryo.

Endogenous Dpp-sfGFP fluorescence and anti-GFP staining (Cy3-conjugated secondary antibody), combined with pMad co-staining in the (A-A''') side view or (B-B' ' ') dorsal view of stage 14 *Drosophila* embryos. (C-C' ' ') Brain with attached leg imaginal discs and (D-D''') eye-antennal imaginal discs.

Development • Supplementary information

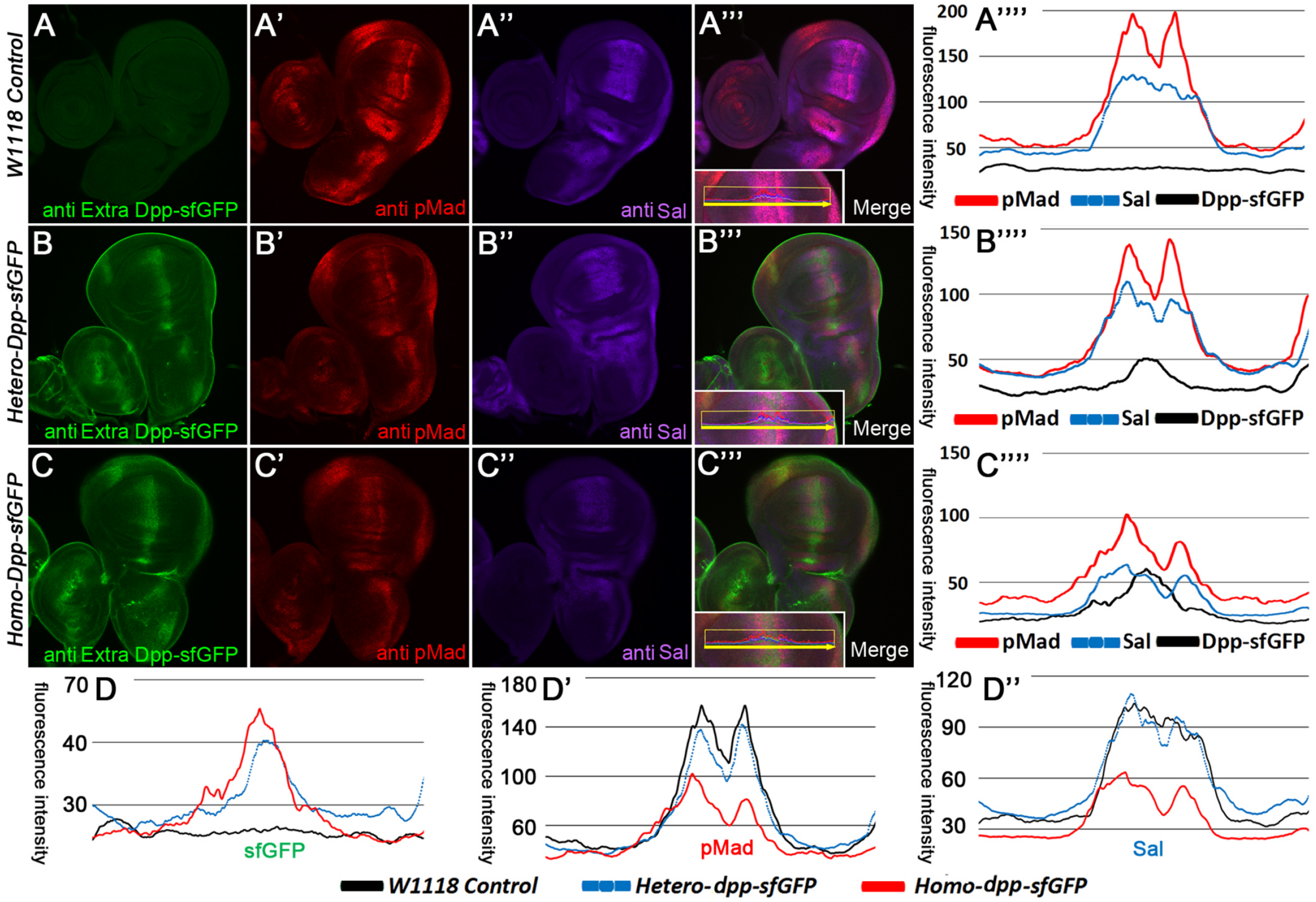

Fig. S4. Signaling activity comparison of hetero/homo-Dpp-sfGFP and wild-type Dpp. (A-C'''') Red, blue, and black lines denote pMad, Sal, and Dpp-sfGFP signals, respectively. Red lines represent pMad staining, blue lines indicate Sal expression, and black lines show Dpp-sfGFP levels. (D-D''') Comparison of fluorescence intensity among different groups for Dpp-sfGFP, pMad, and Sal. The black lines represent them in *W*^{1118} control, blue lines represents them in heterozygous genotype, and red lines show as them in homozygous genotype.

Development • Supplementary information

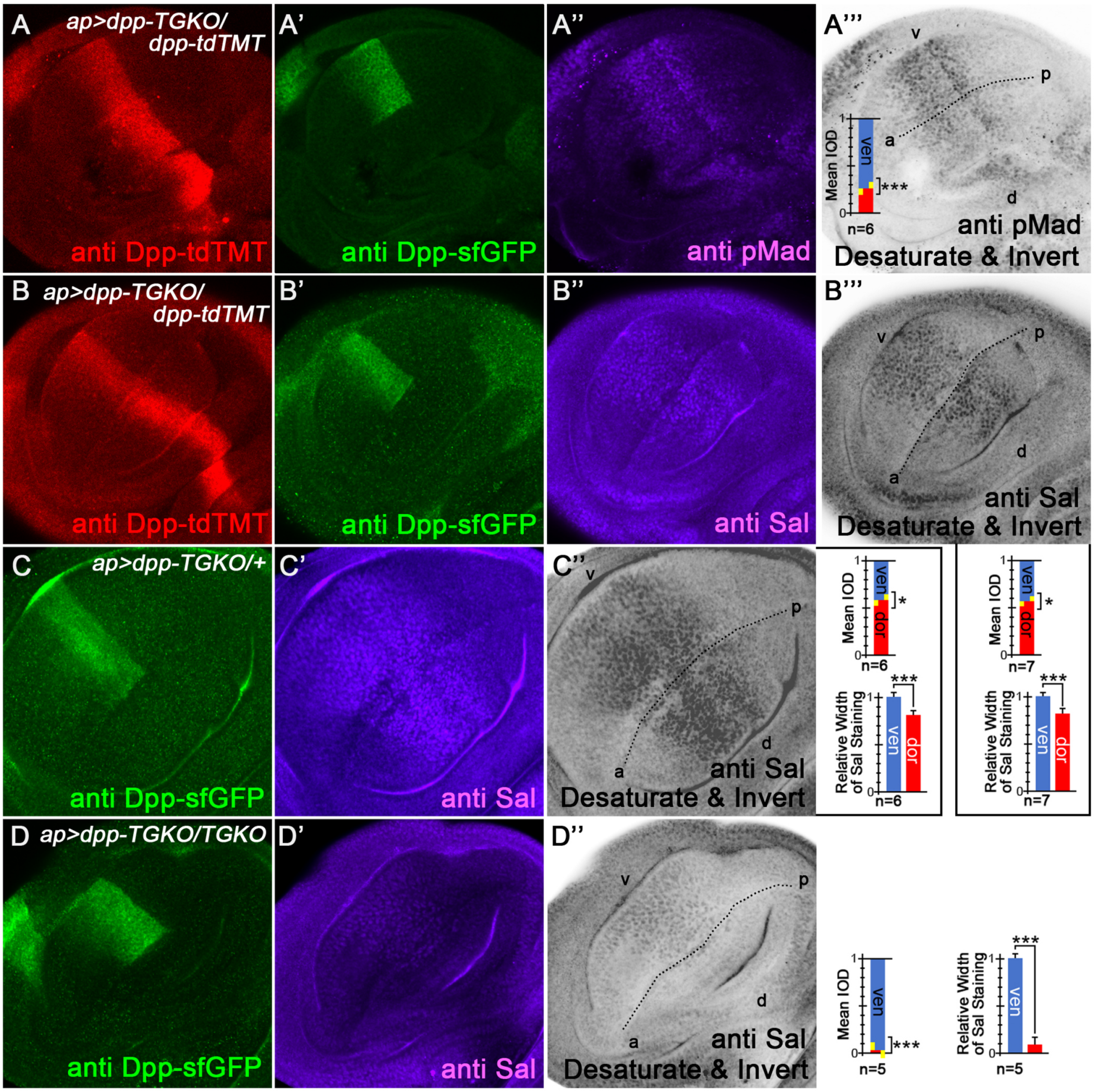

Fig. S5. Sal staining in TGKO hetero/homo-knockout groups.

(A) Another replicate of TGKO hetero-knockout in *ap>TGKO/dpp-tdTMT* background with pMad staining, and (B) with Sal staining. (C) Sal staining in *ap>hetero-TGKO* background and (D) *homo-TGKO* group. Quantification of pouch area and the width of Sal expressive regions.

Development • Supplementary information

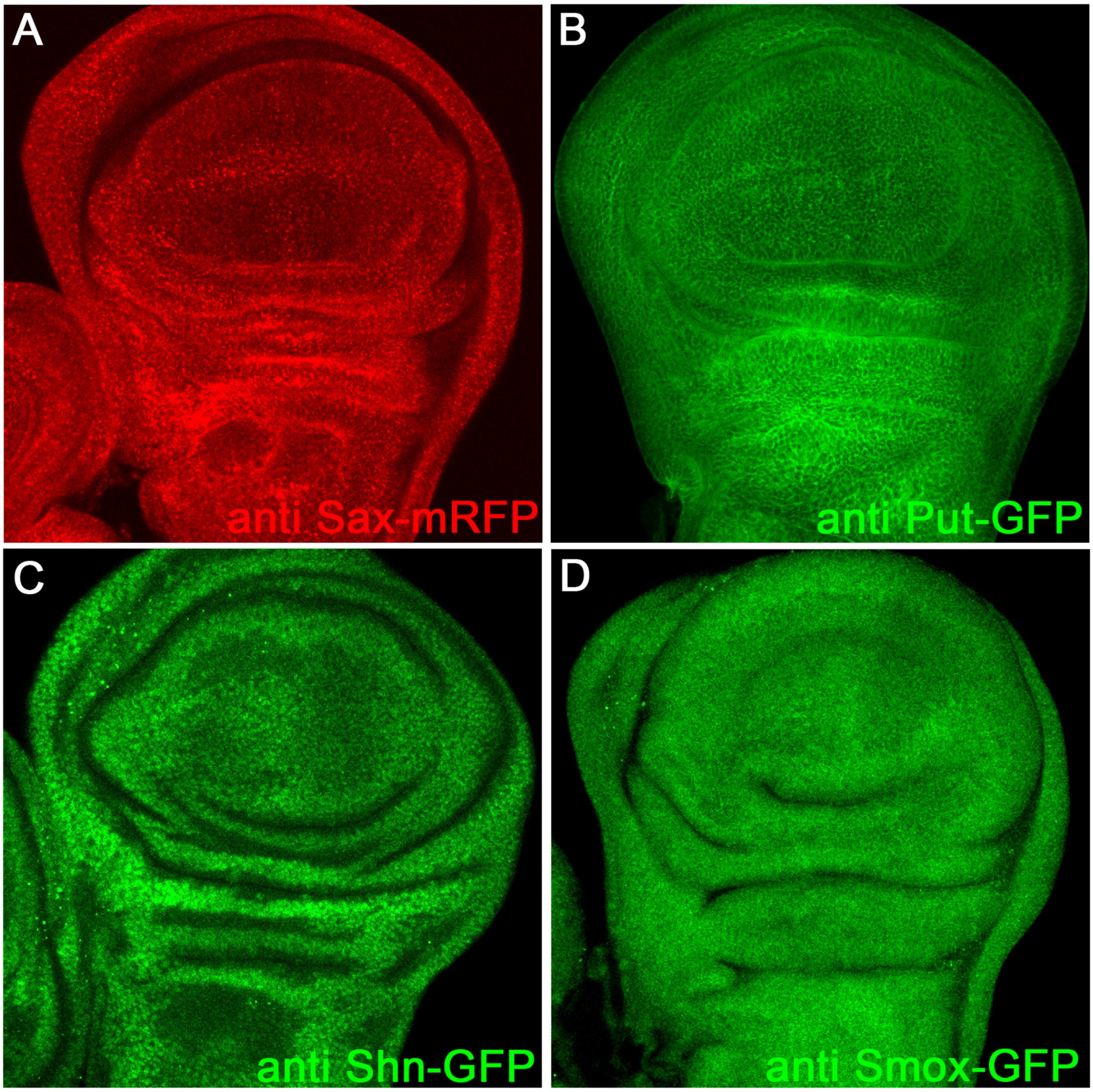

Fig. S6. Expression patterns of other tagging in fly strains.

Wing disc expression patterns of (A) sax-mRFP and (B) punt-YFP (Venus), two endogenously tagged strains generated in this study. (C) *shn-GFP* and (D) *smox-GFP* fluo-GFP lines were obtained from BDSC.

Development • Supplementary information

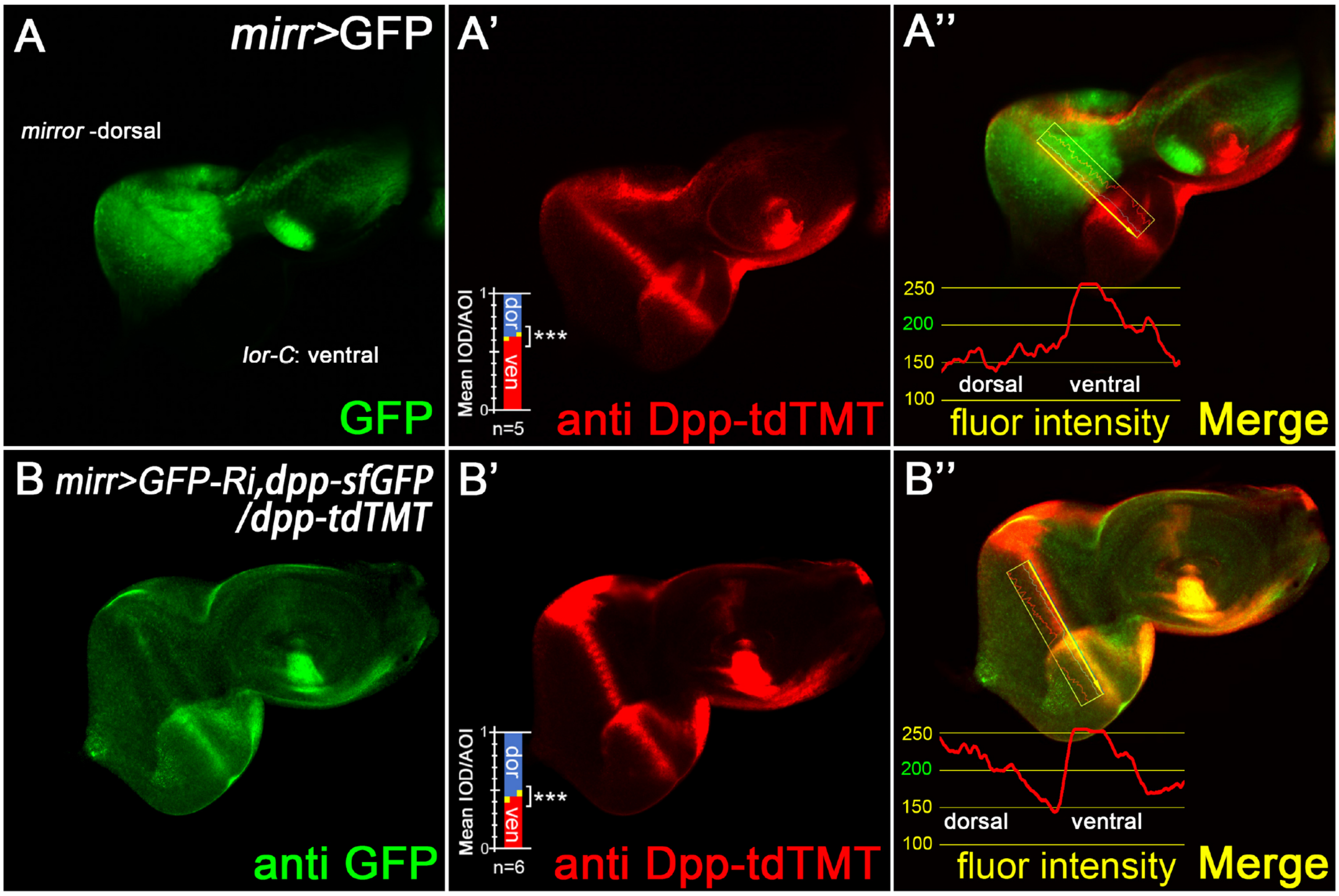

Fig. S7. The feedback mechanism was conserved in the eye imaginal disc.
(A) *mirr>GFP* indicates the Gal4 expression region; (A ') Dpp-tdTMT in the imaginal eye disc; (A ' ') merged image. (B) *mirr>GFP-RNAi* mediated *Dpp-sfGFP* knockdown, leads to Dpp-tdTMT increased in dorsal region (B'); (B'') merged image.

Development • Supplementary information

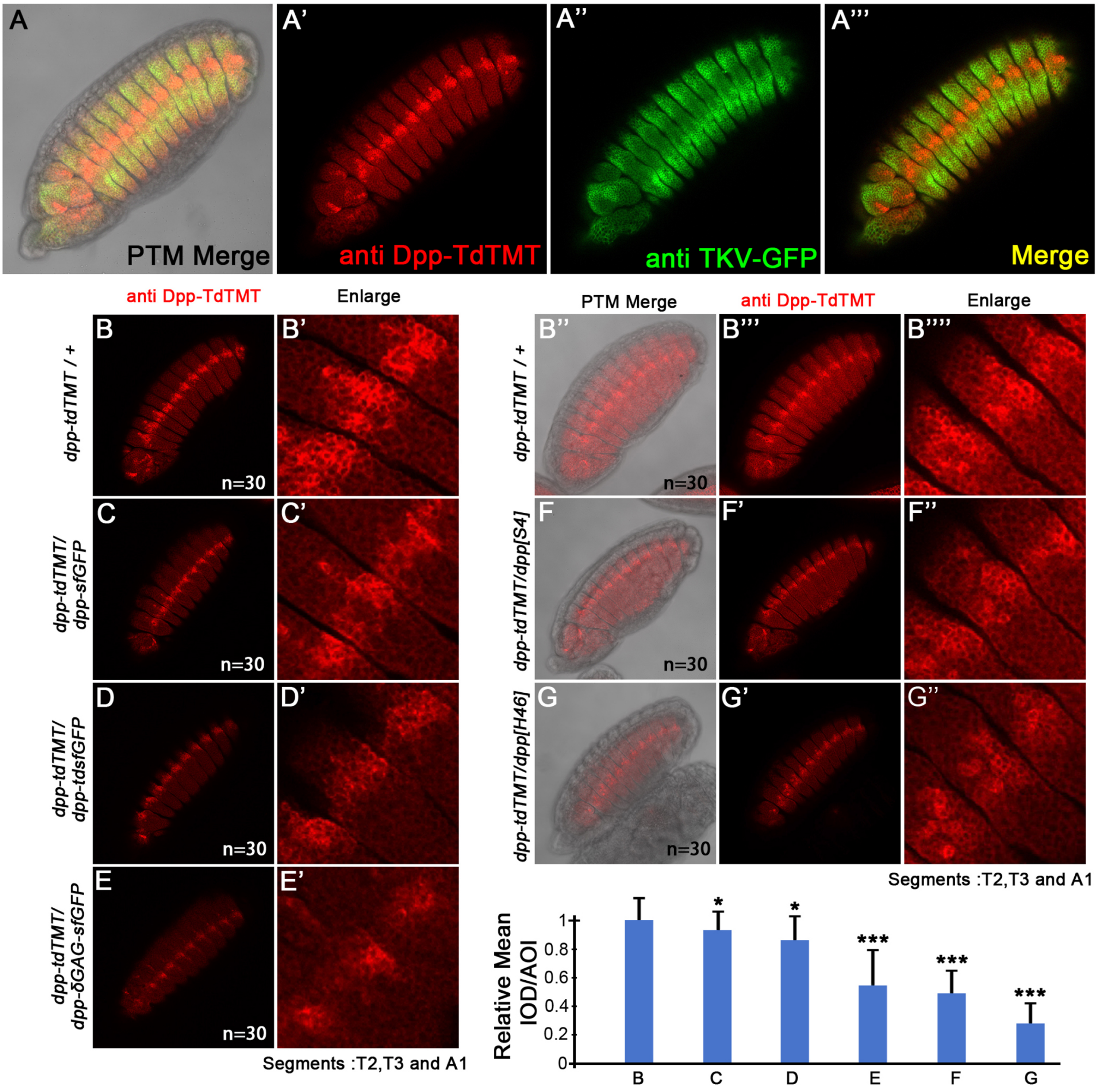

Fig. S8. Analysis of feedback regulation and allelic compensation during embryogenesis. (A) Dpp-tdTMT and Tkv-GFP staining in stage 14 *Drosophila* embryos. *UAS/Gal4*-mediated *RNAi* or overexpression within 0–10 hours post-fertilization yielded suboptimal effects. Therefore, we used hybridization between fixed alleles and alleles of different activation strengths to determine whether there was a dose compensation effect, inferring the presence of a transcriptional feedback regulatory mechanism similar to that in imaginal discs. Unfortunately, in the (A) control

group *dpp-tdTMT/+*, compared with (B) *dpp-tdTMT/dpp-sfGFP*, (C) *dpp-tdTMT/dpp-tdsfGFP*, and (D) *dpp-tdTMT/dpp-δ-GAG-sfGFP*, all showed decreased Dpp-tdTMT expression rather than increased. These three *dpp* lines are modified strains with weaker ligand activation than wild-type *dpp*. The *tdsfGFP* carries double *sfGFP* in linear, and *δ-GAG* line knocks out seven amino acid residues (RRPTRRK) in the HSPG binding domain, which located downstream of the C-terminal furin cleavage sites. We also tested two additional weak alleles: the expression of tdTMT after crossing with *dpp[S4]* and *dpp[H46]*. The results indicated that the two alleles cannot compensate for dosage during embryonic development, and there appears to be no transcriptional feedback mechanism in embryos, leading to dose-dependent lethality due to haploinsufficiency.

Development • Supplementary information

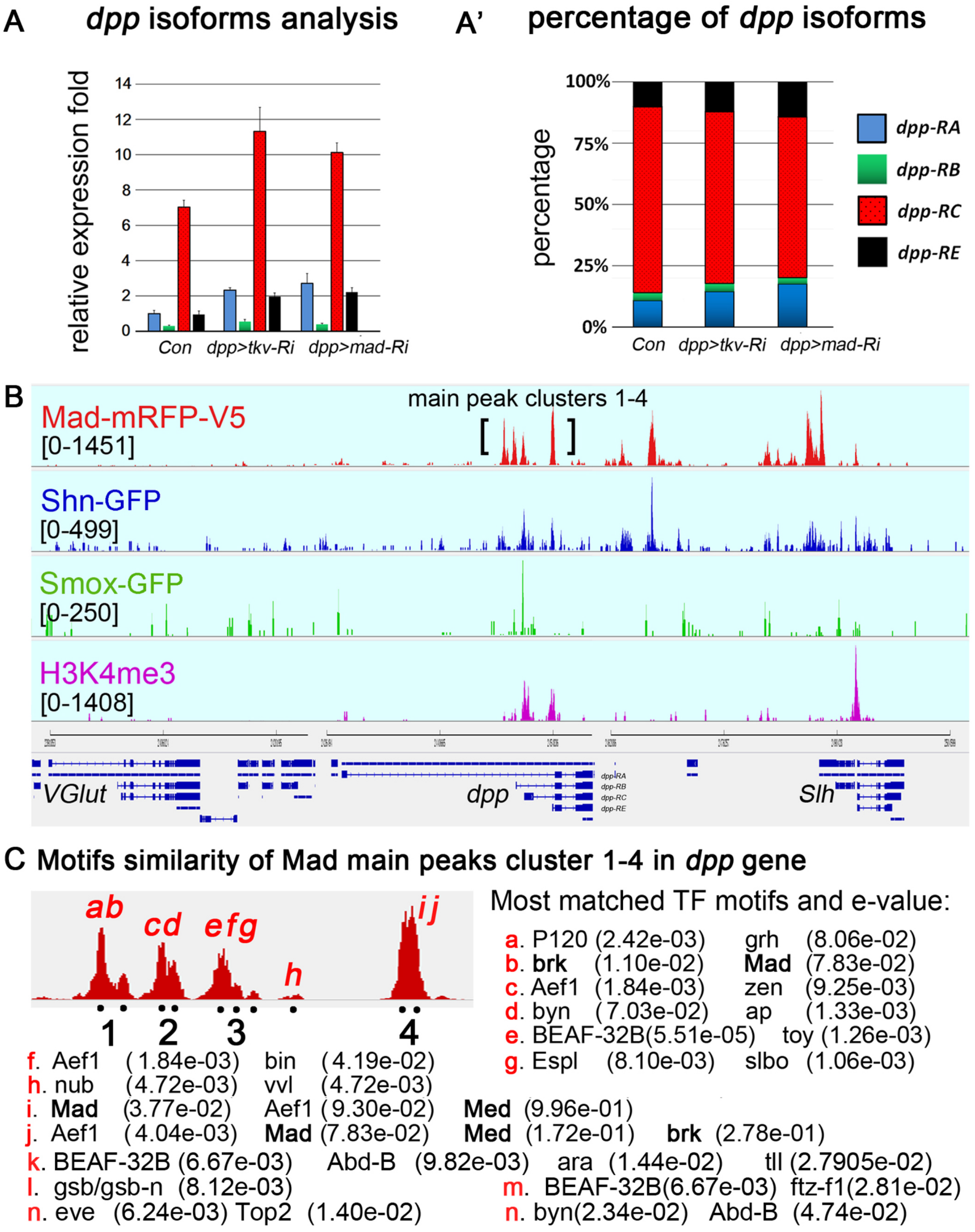

Fig. S9. Analysis the expression of different *dpp* isoforms and whole disc sample CUT&Tag motifs analysis.

(A) Relative expression level of *dpp-RA*, *RB*, *RC*, and *RE* under normal conditions, *dpp>tkv-RNAi,* and *dpp>mad-RNAi* conditions; (A') Expression percentage of *dpp-RA*, *RB*, *RC*, and *RE*. (B) CUT&Tag peak clusters 1–4 (peaks *a*–*j*) in wing discs. (C) Motif prediction for main peaks *a* to *j* using the STAMP online tool (showing partial top-score results; segments of 200 bp in length were selected for analysis, centered on CUT&Tag peaks).

Development • Supplementary information

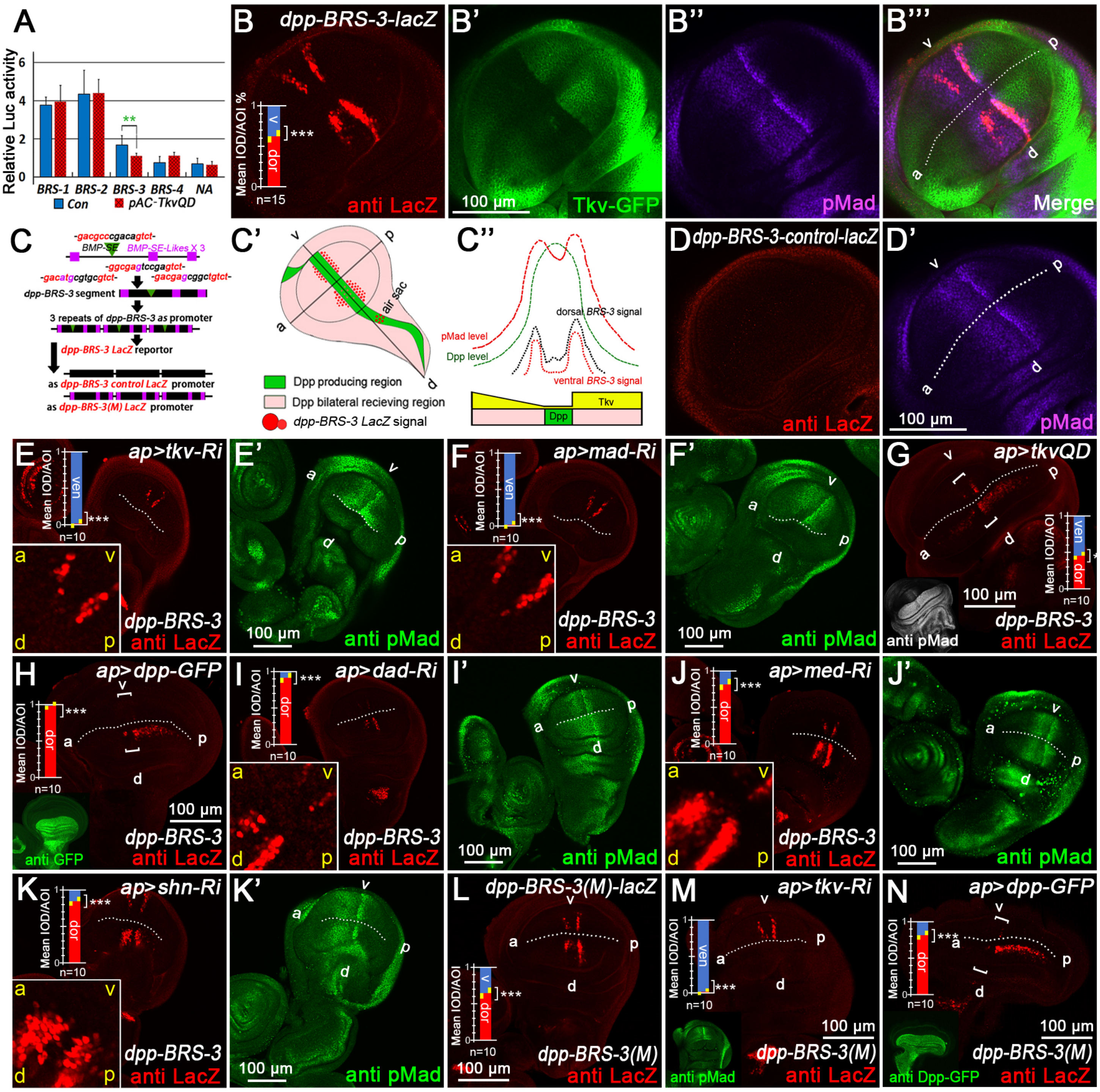

Fig. S10. The dpp-BRS-3 fragment (shv region) exhibits bidirectional responsiveness to Dpp/BMP signaling.

(A) Luciferase assay identification for *dpp-BRS-1* to *4* responses to overexpress TkvQD, showing that only *BRS-3* is subject to negative feedback. (B) LacZ staining for *dpp-BRS-3-lacZ*, together with (B') Tkv-sfGFP and (B'') pMad staining. (C) The scheme for how to generating *dpp-BRS-3-lacZ, dpp-BRS-3-control-lacZ,* and *dpp-BRS-3(M)-lacZ* with *BMP-SE*-removed. (C') The pattern graph for *dpp-BRS-3-lacZ* and (C'') its expression curve

Development • Supplementary information

signaling. Other BMP-SE complex components, including (J) *ap>med-RNAi* and (K) *ap>shn-RNAi*, upregulated LacZ expression. Here, we also examined (L) *dpp-BRS-3(M)-lacZ* with (M) *ap>tkv-RNAi* and (L) *ap>dpp-GFP*, which show similar staining compared with *ap>dpp-GFP*. However, in the *ap>dpp-GFP* group, LacZ signaling seemed more activated than in the *dpp-BRS-3* group may due to the loss of the *BMP-SE* repression site. Additionally, the ventral region pattern strips were not entire repressed compared with the *dpp-BRS-3 group* (indicated region were pointed with white bracket).

Development • Supplementary information

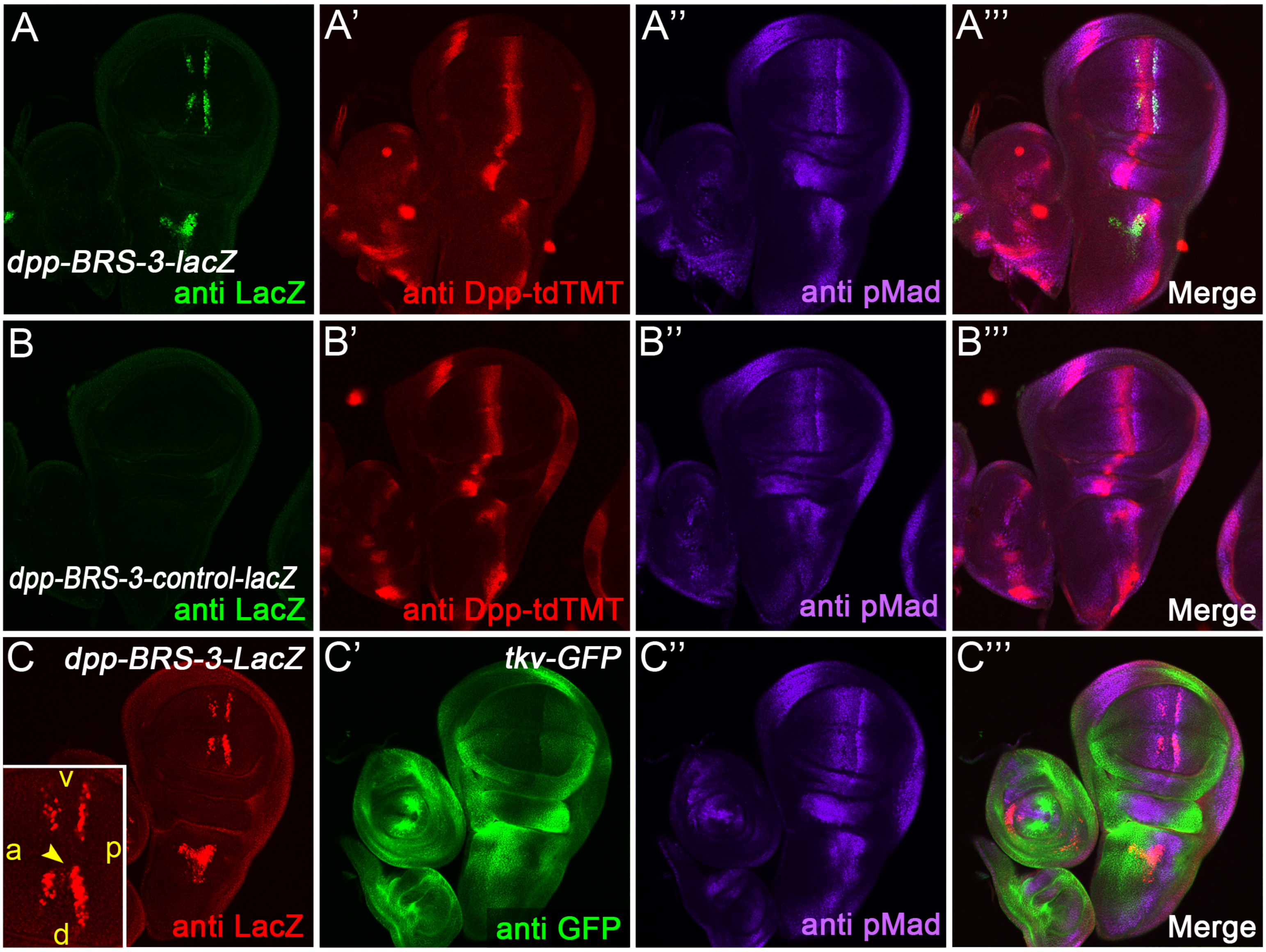

Fig. S11. Entire wing imaginal disc figures of *dpp-BRS-3-lacZ* reporter. (A-A''') LacZ staining of *dpp-BRS-3*-LacZ, together with Dpp-tdTMT and pMad staining. *dpp-BRS-3*-LacZ is expressed on both sides of the Dpp-producing region in the wing disc pouch and in the air sac. (B-B''') LacZ staining of *dpp-BRS-3-control*-LacZ, which deletes all four *BMP-SE*-related motifs in *BRS-3* fragment. (C-C''') Enlarged images of Tkv-sfGFP, *dpp-BRS-3*-LacZ, and pMad staining. Note the weak expression of *dpp-BRS-3*-LacZ in the dorsal region where *dpp* expressed (yellow arrowhead).

Development • Supplementary information

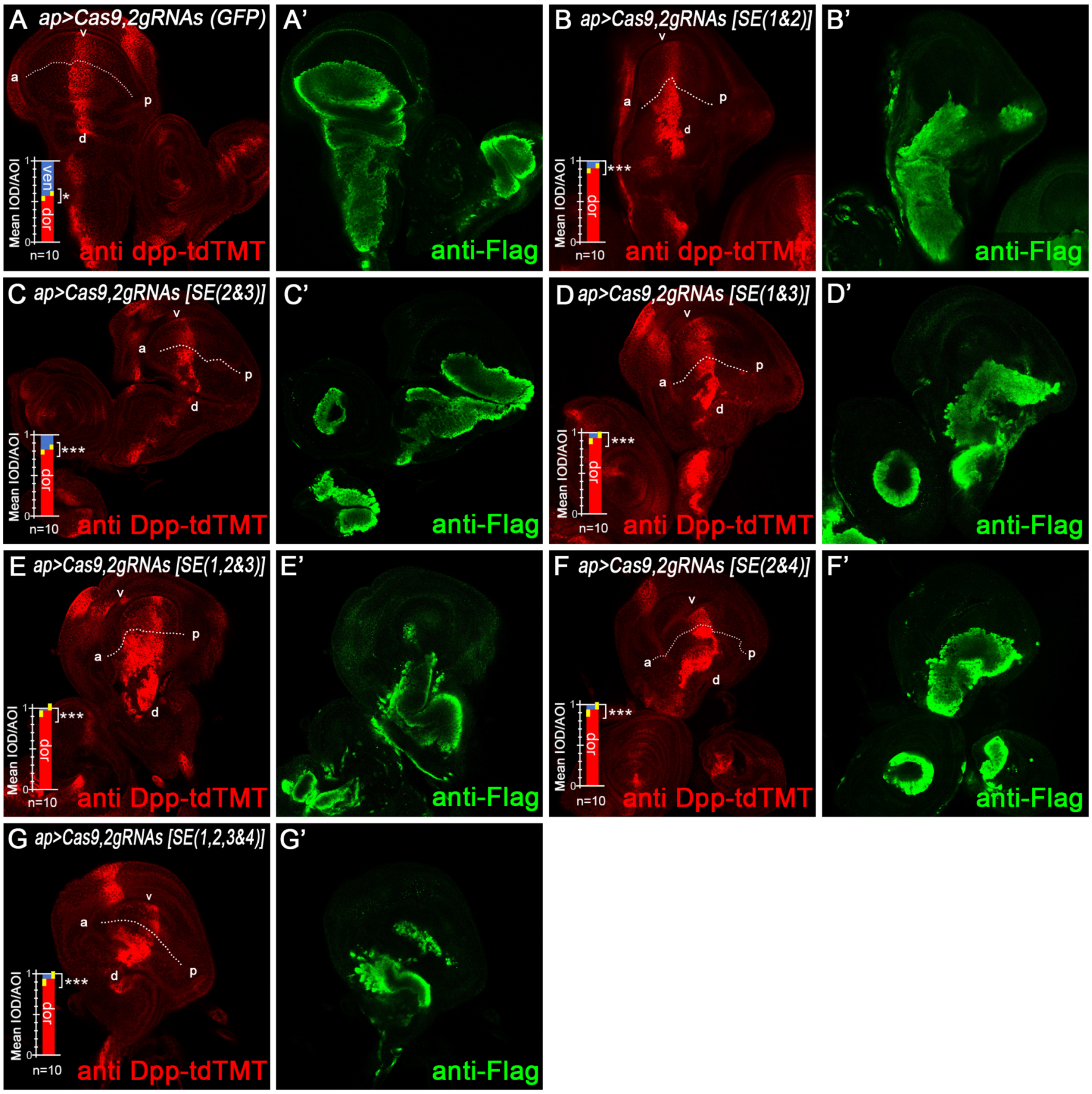

Fig. S12. Dpp-tdTMT expression following CRISPR/Cas9-mediated deletion of combined *BMP-SE* sites.

(A) Control used *gRNAs* against the *GFP* sequence. Combination of (B) *gRNAs [BMP-SE(1&2)]*, (C) *gRNAs [BMP-SE(2&3)]*, (D) *gRNAs[BMP-SE(1&3)]*, (E) *gRNAs [BMP-SE(1, 2&3)]*, (F) *gRNAs [BMP-SE(2&4)]*, and (G) combination of *gRNAs [BMP-SE(1,2,3&4)]. Overall,* the cross-site knockouts globally induced Dpp-tdTMT upregulation.

Development • Supplementary information

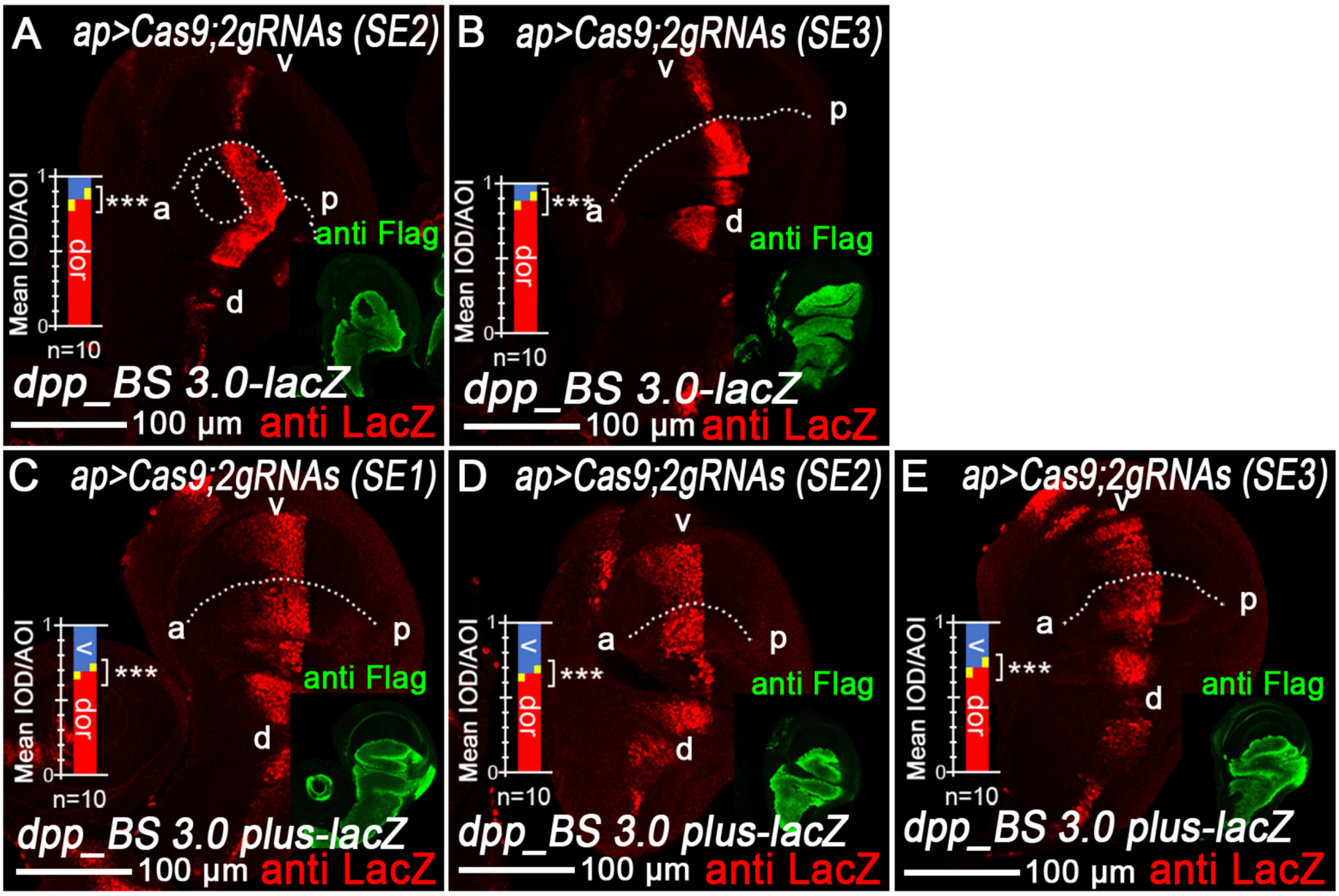

Fig. S13. LacZ expression under *BMP-SE* sites destructed *via* CRISPR/Cas9 system.

Useing the CRISPR/Cas9 system to assess the effect of *BMP-SE* on the (A) *gRNAs* against *SE2* and (B) *gRNAs* against *SE3* in *dpp-BS-3.0-lacZ* strain; *and* and (C) *gRNAs* against *SE1,* (D) *gRNAs* against *SE2*, and (C) *gRNAs* against *SE3* in *dpp-BS-3.0-plus-lacZ* groups. All of them show upregulated in LacZ staining.

Development • Supplementary information

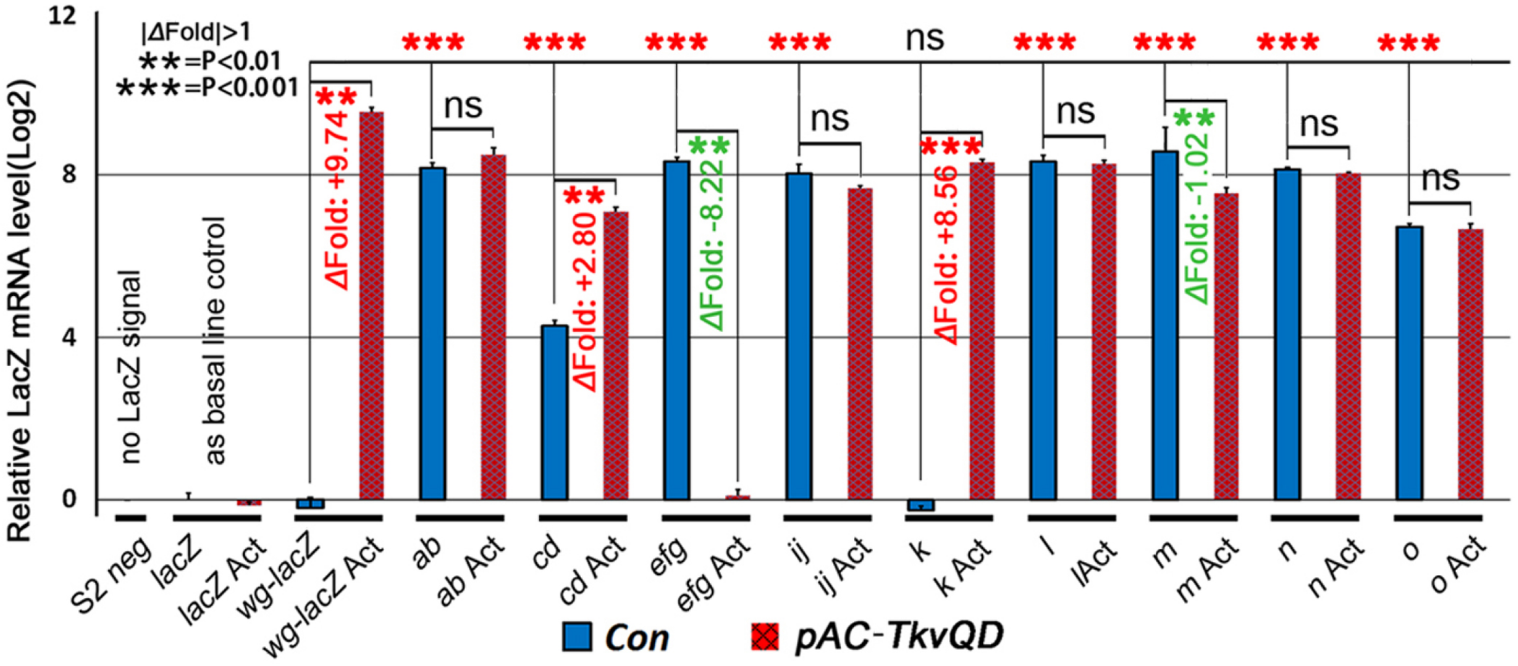

Fig. S14. The signaling responding test for the CUT&Tag related segments (shv and Hin) by *in vitro* reporters RT-qPCR analysis.

RT-qPCR results for relative lacZ mRNA levels for fragments *a* to *o*, with the baseline of the Y-axis normalized to the null *lacZ* group. The data indicated that fragment *cd* and *k* are positive response for Dpp/BMP signaling, while fragment *efg* and *m* are negative responding. RT-qPCR analysis of relative lacZ mRNA levels (fragments *a*-*o*) showed that fragments *cd* and *k* exhibited positive responses to Dpp/BMP signaling, whereas fragments *efg* and *m* were negative feedback to signaling. Data were normalized to the null *lacZ* control group (Y-axis baseline normalized).

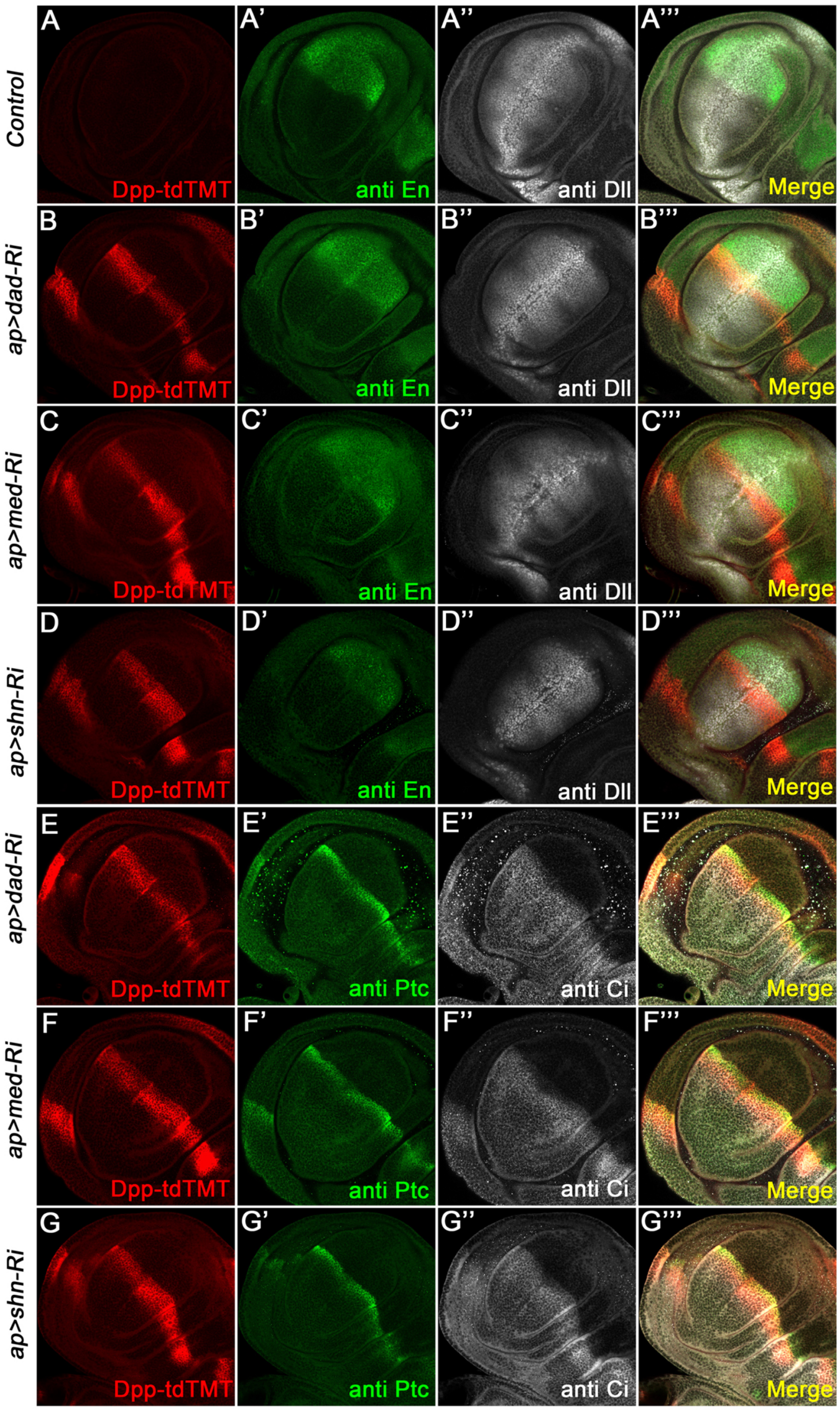

Fig. S15. Dpp/BMP feedback regulation of *dpp* transcription is independent of Hedgehog (Hh), Wingless (Wg), and Engrailed (En) pathways. (A-A''') w^{1118} control; Distal-less(Dll) and Engrailed(En) staining were invariable in (B-B''')*ap>dad-RNAi*, (C-C''')*ap>med-RNAi*, and(D-D''') *ap>shn-RNAi* samples; Patch(Ptc) and Cubitus interruptus(Ci) staining were unchanged in (E-E''') *ap>dad-RNAi*, (F-F''') *ap>med-RNAi*, and (G-G''') *ap>shn-RNAi* groups.

Development • Supplementary information

Table S1. Detailed genotypes for the figures

Figure	Genotype
Fig. 1B,D-D‴	*Dpp-sfGFP/Dpp-sfGFP;+/+*
Fig. 1C-C‴	*Dpp-sfGFP/+;+/+*
Fig. 1E-E″,H	*dpp-TGKO, tub-Gal80^{ts}/ap-Gal4; UAS-flippase/+* (hetero-knockout)
Fig. 1F-F″,I	*dpp-TGKO, tub-Gal80^{ts}/dpp-TGKO, ap-Gal4; UAS-flppase/+* (homo-knockout)
Fig. 1G	*dpp-TGKO/dpp-TGKO;+/+*
Fig. 2A,A′	*dpp-tdTMT/dpp-sfGFP*
Fig. 2B,B′	*dpp-tdTMT, ap-Gal4/+; +/UAS-GFP*
Fig. 2C-C‴	*dpp-sfGFP, tub-Gal80^{ts}/dpp-tdTMT,ap-Gal4; UAS-GFP-RNAi /+*
Fig. 2D-D‴	*dpp-TGKO, tub-Gal80^{ts}/dpp-tdTMT, ap-Gal4; UAS-flippase/+*
Fig. 2E	*dpp-tdTMT, ap-Gal4/tub-Gal80^{ts};+/UAS-luciferase(luc)-RNAi* (as control)
Fig. 2F,F′	*dpp-tdTMT, ap-Gal4/tub-Gal80^{ts}; +/UAS-tkv-RNAi*
Fig. 2G,G′	*dpp-tdTMT, ap-Gal4/tub-Gal80^{ts}; +/UAS-mad-RNAi*
Fig. 2H,H′	*dpp-tdTMT, ap-Gal4/tub-Gal80^{ts}; +/UAS-tkvQD*
Fig. 2I-I″	*dpp-tdTMT, ap-Gal4/tub-Gal80^{ts}; +/UAS-dpp-GFP*
Fig. 3A,A″	*dpp-TGKO, tub-Gal80^{ts}/dpp-tdTMT, ap-Gal4; UAS-flippase/+*
Fig. 3B,B′	*dpp-tdTMT, ap-Gal4/ tub-Gal80^{ts}; + /UAS-tkvQD*
Fig. 3C,C′	*dpp-tdTMT, ap-Gal4/tub-Gal80^{ts}; + /UAS-dpp-GFP*
Fig. 3D,D′	*ap-Gal4/tub-Gal80^{ts}; +/UAS-luc-RNAi* (as control)
Fig. 3E,E′	*dpp-tdTMT, ap-Gal4/tub-Gal80^{ts}; +/UAS-tkv-RNAi*
Fig. 3F,F′	*dpp-tdTMT, ap-Gal4/ tub-Gal80^{ts}; +/UAS-mad-RNAi*
Fig. 3G,G′	*dpp-tdTMT, ap-Gal4/tub-Gal80^{ts}; +/UAS-tkvQD*
Fig. 3H,H‴	*dpp-TGKO, tub-Gal80^{ts}/dpp-LacZ[10638],ap-Gal4; UAS-flippase/+*
Fig. 3I,I″	*dpp-LacZ[10638], ap-Gal4/tub-Gal80^{ts}; +/UAS-dpp-GFP*

Fig. 3J,J′	*dpp_BS 3.0-lacZ, ap-Gal4/tub-Gal80^{ts}; +/UAS-luc-RNAi* (as control)
Fig. 3K,K′	*dpp_BS 3.0-lacZ, ap-Gal4/ tub-Gal80^{ts}; +/UAS-tkv-RNAi*
Fig. 3L,L′	*dpp_BS 3.0-lacZ, ap-Gal4/ tub-Gal80^{ts}; +/UAS-mad-RNAi*
Fig. 3M,M′	*dpp_BS 3.0-lacZ, ap-Gal4/tub-Gal80^{ts}; +/UAS-tkvQD*
Fig. 3N,N′	*dpp_BS 3.0-lacZ, ap-Gal4/tub-Gal80^{ts}; +/UAS-dpp-GFP*
Fig. 4C,C′	*dpp-tdTMT,ap-Gal4/+; +/UAS-luc-RNAi* (as control)
Fig. 4D,D′	*dpp-tdTMT, ap-Gal4/ +; + /UAS-dad-RNAi*
Fig. 4E,E′	*dpp-tdTMT, ap-Gal4/+; +/UAS-med-RNAi*
Fig. 4F,F′	*dpp-tdTMT, ap-Gal4/+; +/UAS-shn-RNAi*
Fig. 5B,B′	*dpp-tdTMT, ap-Gal4/+; +/UAS-Flag-Cas9-dU6-dual-gRNAs (GFP)* (as control)
Fig. 5C-C″	*dpp-tdTMT, ap-Gal4/+; +/UAS-Flag-Cas9-dU6-dual-gRNAs (BMP-SE1)*
Fig. 5D-D″	*dpp-tdTMT, ap-Gal4/+; +/UAS-Flag-Cas9-dU6-dual-gRNAs (BMP-SE1us)*
Fig. 5E,E′	*dpp-tdTMT, ap-Gal4/UAS-Flag-Cas9-dU6-dual-gRNAs (BMP-SE2); +/+*
Fig. 5F,F′	*dpp-tdTMT, ap-Gal4/+; +/UAS-Flag-Cas9-dU6-dual-gRNAs (BMP-SE3)*
Fig. 5G,G′	*dpp-tdTMT, ap-Gal4/UAS-Flag-Cas9-dU6-dual-gRNAs (BMP-SE4); +/+*
Fig. 6B,B′	*+/+;dpp_BS 3.0-plus-lacZ/dpp_BS 3.0-plus-lacZ*
Fig. 6C,C′	*+/+;dpp_BS 3.0-plus (M)-lacZ/dpp_BS 3.0-plus (M)-lacZ*
Fig. 6D	*ap-Gal4/tub-Gal80^{ts} ; dpp_BS 3.0-plus-lacZ/ UAS-luc-RNAi*
Fig. 6E	*ap-Gal4/tub-Gal80^{ts} ; dpp_BS 3.0-plus-lacZ/ UAS-mad-RNAi*
Fig. 6F	*ap-Gal4/+; dpp_BS 3.0-plus-lacZ/ UAS-med-RNAi*
Fig. 6G	*ap-Gal4/+; dpp_BS 3.0-plus-lacZ/ UAS-shn-RNAi*
Fig. 6H	*ap-Gal4/tub-Gal80^{ts}; dpp_BS 3.0-plus-lacZ/ UAS-tkv-RNAi*
Fig. 6I	*ap-Gal4/tub-Gal80^{ts}; dpp_BS 3.0-plus-lacZ/ UAS-dpp-GFP*
Fig. 7A-A″,C,C′ left side	*Mad-mRFP-V5/Mad-mRFP-V5; +/dpp-Gal4, UAS-CD8-GFP* (sorted GFP+ cells as control sample for CUT&Tag assay)
Fig. 7B-B″,C,C′	*Mad-mRFP-V5/Mad-mRFP-V5; UAS-dpp/dpp-Gal4, UAS-*

right side	*CD8-GFP* (sorted GFP+ cells as BMP-activating sample for CUT&Tag assay)
Fig. S3A-D‴	*Dpp-sfGFP/Dpp-sfGFP; +/+*
Fig. S4A-A″″	*+/+;+/+ (W1118)*
Fig. S4B-B″″	*dpp-sfGFP/+;+/+*
Fig. S4C-C″″	*dpp-sfGFP/dpp-sfGFP;+/+*
Fig. S5A-B‴	*dpp-TGKO, tub-Gal80^{ts}/ dpp-tdTMT, ap-Gal4; UAS-flippase/+*
Fig. S5C-C″	*dpp-TGKO, tub-Gal80^{ts}/+, ap-Gal4; UAS-flippase/+* (hetero-knockout)
Fig. S5D-D″	*dpp-TGKO, tub-Gal80^{ts}/dpp-TGKO, ap-Gal4; UAS-flippase/+* (homo-knockout)
Fig. S6A	*sax-mRFP-V5/sax-mRFP-V5;+/+*
Fig. S6B	*+/+;put-YFP (Venus)/put-YFP (Venus)*
Fig. S6C	*shn-GFP/shn-GFP;+/+*
Fig. S6D	*smox-GFP/smox-GFP;+/+;+/+*
Fig. S7A-A″	*dpp-tdTMT, Tub-Gal80^{ts}/UAS-GFP; +/mirr-Gal4*
Fig. S7B-B″	*dpp-sfGFP, tub-Gal80^{ts}/dpp-tdTMT ; UAS-GFP-RNAi /mirr-Gal4*
Fig. S8A-A‴	*dpp-tdTMT/+;+/tkv-GFP*
Fig. S8B-B″″	*dpp-tdTMT/+;+/+*
Fig. S8C,C′	*dpp-tdTMT/dpp-sfGFP;+/+*
Fig. S8D,D′	*dpp-tdTMT/dpp-tdsfGFP;+/+*
Fig. S8E,E′	*dpp-tdTMT/dpp-δGAG-sfGFP;+/+*
Fig. S8F,F′	*dpp-tdTMT/dpp[S4];+/+*
Fig. S8G,G′	*dpp-tdTMT/dpp[H46];+/+*
Fig. S10B-B‴	*+/+;dpp-BRS-3-lacZ /dpp-BRS-3-lacZ*
Fig. S10D,D′	*+/+;dpp-BRS-3-control-lacZ /dpp-BRS-3-control-lacZ*
Fig. S10E,E′	*ap-Gal4/tub-Gal80^{ts};dpp-BRS-3-lacZ/UAS-tkv-RNAi*
Fig. S10F,F′	*ap-Gal4/tub-Gal80^{ts};dpp-BRS-3-lacZ/UAS-mad-RNAi*
Fig. S10G	*ap-Gal4/tub-Gal80^{ts};dpp-BRS-3-lacZ/UAS-tkvQD*
Fig. S10H	*ap-Gal4/tub-Gal80^{ts};dpp-BRS-3-lacZ/UAS-dpp-GFP*

Development • Supplementary information

Fig. S10I and I′	*ap-Gal4/+;dpp-BRS-3-lacZ/UAS-dad-RNAi*
Fig. S10J,J′	*ap-Gal4/+;dpp-BRS-3-lacZ/UAS-med-RNAi*
Fig. S10 K,K′	*ap-Gal4/+;dpp-BRS-3-lacZ/UAS-shn-RNAi*
Fig. S10L	*+/+;dpp-BRS-3 (M)-lacZ/dpp-BRS-3 (M)-lacZ*
Fig. S10M	*ap-Gal4/tub-Gal80^{ts};dpp-BRS-3 (M)-lacZ/UAS-tkv-RNAi*
Fig. S10N	*ap-Gal4/tub-Gal80^{ts};dpp-BRS-3 (M)-lacZ/UAS-dpp-GFP*
Fig. S11A-A‴	*dpp-tdTMT/+;+/dpp-BRS-3-lacZ*
Fig. S11B-B‴	*dpp-tdTMT/+;+/dpp-BRS-3-control-lacZ*
Fig. S11C-C″	*tkv-GFP/+;+/dpp-BRS-3-lacZ*
Fig. S12A,A′	*dpp-tdTMT,ap-Gal4/+ ; +/UAS-Flag-Cas9-dU6-dual-gRNAs (GFP)*
Fig. S12B,B′	*dpp-tdTMT,ap-Gal4/UAS-Flag-Cas9-dU6-dual-gRNAs (BMP-SE2); +/UAS-Flag-Cas9-dU6-dual-gRNAs (BMP-SE1)*
Fig. S12C,C′	*dpp-tdTMT,ap-Gal4/UAS-Flag-Cas9-dU6-dual-gRNAs (BMP-SE2); +/UAS-Flag-Cas9-dU6-dual-gRNAs (BMP-SE3)*
Fig. S12D,D′	*dpp-tdTMT,ap-Gal4/+; +/UAS-Flag-Cas9-dU6-quadra-gRNAs (BMP-SE1&3)*
Fig. S12E,E′	*dpp-tdTMT,ap-Gal4/UAS-Flag-Cas9-dU6-dual-gRNAs (BMP-SE2); +/UAS-Flag-Cas9-dU6-quadra-gRNAs (BMP-SE1&3)*
Fig. S12F,F′	*dpp-tdTMT,ap-Gal4/UAS-Flag-Cas9-dU6-quadra-gRNAs (BMP-SE2&4); +/+*
Fig. S12F,F′	*dpp-tdTMT,ap-Gal4/UAS-Flag-Cas9-dU6-quadra-gRNAs (BMP-SE2&4); +/UAS-Flag-Cas9-dU6-quadra-gRNAs (BMP-SE1&3)*
Fig. S13A	*ap-Gal4/+; dpp_BS 3.0-lacZ/UAS-Flag-Cas9-dU6-dual-gRNAs (BMP-SE2)*
Fig. S13B	*ap-Gal4/+; dpp_BS 3.0-lacZ/UAS-Flag-Cas9-dU6-dual-gRNAs (BMP-SE3)*
Fig. S13C	*ap-Gal4/+; dpp_BS 3.0-plus-lacZ/ UAS-Flag-Cas9-dU6-dual-gRNAs (BMP-SE1)*
Fig. S13D	*ap-Gal4/+; dpp_BS 3.0-plus-lacZ/ UAS-Flag-Cas9-dU6-dual-gRNAs (BMP-SE2)*

Fig. S13E	*ap-Gal4/+; dpp_BS 3.0-plus-lacZ/ UAS-Flag-Cas9-dU6-dual-gRNAs (BMP-SE3)*
Fig. S15A-A′′′	*+/+;+/+ (W1118)*
Fig. S15B-B′′′,E-E′′′	*dpp-tdTMT, ap-Gal4/+; +/UAS-dad-RNAi*
Fig. S15C-C′′′,F-F′′′	*dpp-tdTMT, ap-Gal4/+; +/UAS-med-RNAi*
Fig. S15D-D′′′,G-G′′′	*dpp-tdTMT, ap-Gal4/+; +/UAS-shn-RNAi*

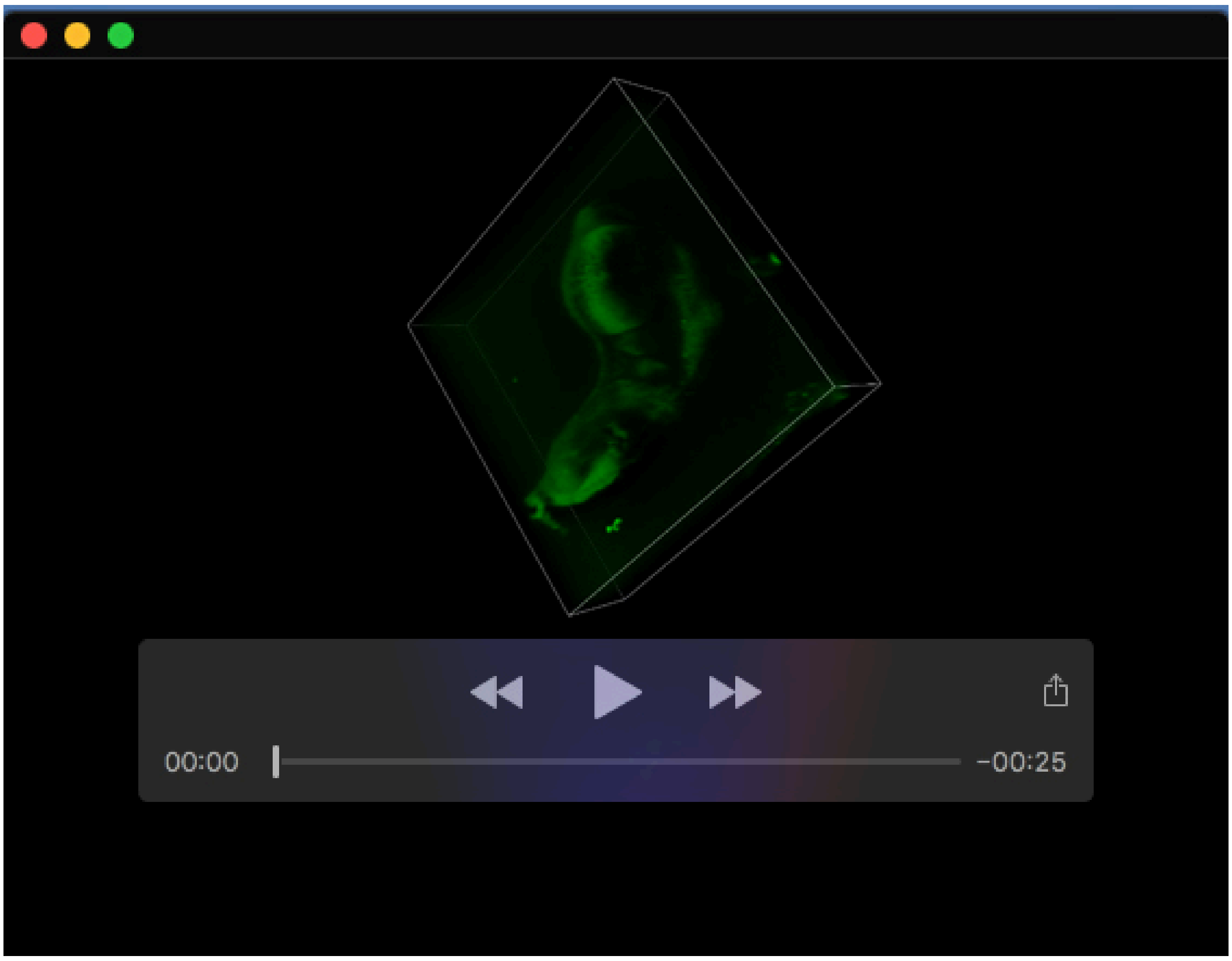

Movie 1. Living image of Dpp-sfGFP in 3rd instar larvae wing imaginal disc. This movie recorded the living imaging data of Dpp-sfGFP expression pattern in the third instar larvae wing imaginal disc, without fixation and staining.

Development • Supplementary information

Supplementary Materials and Methods

1.Genetic strategies employed in this Study: gene editing and transgenic fly construction

1.1. Complete sequence of the sfGFP tagging plasmid for Homologous Recombination

TTTCATCCCCGATATGCACCACCGGGTAAAGTTCACGGGAGACTTTATCTGACAGCAGACGTGCACT
GGCCAGGGGGATCACCATCCGTCGCCCCGGCGTGTCAATAATATCACTCTGTACATCCACAAACAG
ACGATAACGGCTCTCTCTTTTATAGGTGTAAACCTTAAACTGCCGTACGTATAGGCTGCGCAACTGTT
GGGAAGGGCGATCGGTGCGGGCCTCTTCGCTATTACGCCAGCTGGCGAAAGGGGGATGTGCTGCA
AGGCGATTAAGTTGGGTAACGCCAGGGTTTTCCCAGTCACGACGTTGTAAAACGACGGCCAGTGAA
TTGTAATACGACTCACTATAGGGCGAATTGAATTTAGCGGCCGCGAATTCGCCCTTGGTGGATCTGG
AGGTT**GGATCC**ggt**CCTAGG**ggt**ACGCGT**ggt**GGTACC** **(MCS:-BamHI-AvrII-MluI-KpnI- for 5 Arm)**
ATGGTGTCCAAGGGCGAGGAGCTGTTCACCGGCGTGGTGCCCATCCTGGTGGAGCTGGATGGCG
ACGTGAACGGCCACAAGTTCAGCGTGCGCGGCGAGGGCGAGGGCGACGCCACCAACGGCAAGC
TGACCCTGAAGTTCATCTGCACCACCGGCAAGCTGCCCGTGCCCTGGCCCACCCTGGTGACCACC
CTGACCTACGGCGTGCAGTGCTTCAGCCGCTACCCCGATCACATGAAGCAGCACGATTTCTTCAAG
AGCGCCATGCCCGAGGGCTACGTGCAGGAGCGCACCATCAGCTTCAAGGATGACGGCACCTACAA
GACCCGCGCCGAGGTGAAGTTCGAGGGCGATACCCTGGTGAACCGCATCGAGCTGAAGGGCATC
GATTTCAAGGAGGATGGCAACATCCTGGGCCACAAGCTGGAGTACAACTTCAACAGCCACAACGTG
TACATCACCGCCGATAAGCAGAAGAACGGCATCAAGGCCAACTTCAAGATCCGCCACAATGTGGAG
GATGGCTCCGTGCAGCTGGCCGATCACTACCAGCAGAACACCCCCATCGGCGACGGCCCAGTGCT
GCTGCCCGATAACCACTACCTGAGCACCCAGAGCGTGCTGTCCAAGGACCCCAACGAGAAGCGCG
ATCACATGGTGCTGCTGGAGTTCGTGACCGCCGCCGGCATCACCCTGGGCATGGATGAGCTGTAC
AAG (sfGFP sequence, Others just instead of it) **AGATCT**ggt**GTCGAC**ggt**GGCGCGCC**tggt**CTCGAG**
(MCS:-BglII-SalI-AscI-XhoI-SpeI- for 3 Arm) GGTTCTGGTGGTTCAGGAGGTTCCAAGGGCGAATT
CGTTTAAACCTGCAGG**ACTAGT**CCCTTTAGTGAGGGTTAATTCTGAGCTTGGCGTAATCATGGTCATA
GCTGTTTCCTGTGTGAAATTGTTATCCGCTCACAATTCCACACAACATACGAGCCGGAAGCATAAAGT
GTAAAGCCTGGGGTGCCTAATGAGTGAGCTAACTCACATTAATTGCGTTGCGCTCACTGCCCGCTTT
CCAGTCGGGAAACCTGTCGTGCCAGCTGCATTAATGAATCGGCCAACGCGCGGGGAGAGGCGGTT
TGCGTATTGGGCGCTCTTCCGCTTCCTCGCTCACTGACTCGCTGCGCTCGGTCGTTCGGCTGCGG
CGAGCGGTATCAGCTCACTCAAAGGCGGTAATACGGTTATCCACAGAATCAGGGGATAACGCAGGA
AAGAACATGTGAGCAAAAGGCCAGCAAAAGGCCAGGAACCGTAAAAAGGCCGCGTTGCTGGCGTT
TTTCCATAGGCTCCGCCCCCCTGACGAGCATCACAAAAATCGACGCTCAAGTCAGAGGTGGCGAAA
CCCGACAGGACTATAAAGATACCAGGCGTTTCCCCCTGGAAGCTCCCTCGTGCGCTCTCCTGTTCC
GACCCTGCCGCTTACCGGATACCTGTCCGCCTTTCTCCCTTCGGGAAGCGTGGCGCTTTCTCATAG
CTCACGCTGTAGGTATCTCAGTTCGGTGTAGGTCGTTCGCTCCAAGCTGGGCTGTGTGCACGAACC
CCCCGTTCAGCCCGACCGCTGCGCCTTATCCGGTAACTATCGTCTTGAGTCCAACCCGGTAAGACA
CGACTTATCGCCACTGGCAGCAGCCACTGGTAACAGGATTAGCAGAGCGAGGTATGTAGGCGGTGC
TACAGAGTTCTTGAAGTGGTGGCCTAACTACGGCTACACTAGAAGAACAGTATTTGGTATCTGCGCT
CTGCTGAAGCCAGTTACCTTCGGAAAAAGAGTTGGTAGCTCTTGATCCGGCAAACAAACCACCGCT
GGTAGCGGTGGTTTTTTTGTTTGCAAGCAGCAGATTACGCGCAGAAAAAAAGGATCTCAAGAAGATC
CTTTGATCTTTTCTACGGGGTCTGACGCTCAGTGGAACGAAAACTCACGTTAAGGGATTTTGGTCAT
GAGACAATAACCCTGATAAATGCTTCAATAATATTGAAAAAGGAAGAGTATGAGTATTCAACATTTCCG
TGTCGCCCTTATTCCCTTTTTTGCGGCATTTTGCCTTCCTGTTTTTGCTCACCCAGAAACGCTGGTG
AAAGTAAAAGATGCTGAAGATCAGTTGGGTGCACGAGTGGGTTACATCGAACTGGATCTCAACAGC
GGTAAGATCCTTGAGAGTTTTCGCCCCGAAGAACGTTTTCCAATGATGAGCACTTTTAAAGTTCTGCT
ATGTGGCGCGGTATTATCCCGTATTGACGCCGGGCAAGAGCAACTCGGTCGCCGCATACACTATTCT
CAGAATGACTTGGTTGAGTACTCACCAGTCACAGAAAAGCATCTTACGGATGGCATGACAGTAAGAG
AATTATGCAGTGCTGCCATAACCATGAGTGATAACACTGCGGCCAACTTACTTCTGACAACGATCGG
AGGACCGAAGGAGCTAACCGCTTTTTTGCACAACATGGGGGATCATGTAACTCGCCTTGATCGTTG
GGAACCGGAGCTGAATGAAGCCATACCAAACGACGAGCGTGACACCACGATGCCTGTAGCAATGG
CAACAACGTTGCGCAAACTATTAACTGGCGAACTACTTACTCTAGCTTCCCGGCAACAATTAATAGAC
TGGATGGAGGCGGATAAAGTTGCAGGACCACTTCTGCGCTCGGCCCTTCCGGCTGGCTGGTTTATT
GCTGATAAATCTGGAGCCGGTGAGCGTGGGTCTCGCGGTATCATTGCAGCACTGGGGCCAGATGGT
AAGCCCTCCCGTATCGTAGTTATCTACACGACGGGGAGTCAGGCAACTATGGATGAACGAAATAGAC
AGATCGCTGAGATAGGTGCCTCACTGATTAAGCATTGGTAACTGTCAGACCAAGTTTACTCATATATA
CTTTAGATTGATTTAAAACTTCATTTTTAATTTAAAAGGATCTAGGTGAAGATCCTTTTTGATAATCTCAT
GAGCGGATACATATTTGAATGTATTTAGAAAAATAAACAAATAGGGGTTCCGCGCACATTTCCCCGAA
AAGTGCCACCTGATGCGGTGTGAAATACCGCACAGATGCGTAAGGAGAAAATACCGCATCAGGAAA
TTGTAAGCGTTAATAATTCAGAAGAACTCGTCAAGAAGGCGATAGAAGGCGATGCGCTGCGAATCGG
GAGCGGCGATACCGTAAAGCACGAGGAAGCGGTCAGCCCATTCGCCGCCAAGCTCTTCAGCAATAT
CACGGGTAGCCAACGCTATGTCCTGATAGCGGTCCGCCACACCCAGCCGGCCACAGTCGATGAAT

Development • Supplementary information

CCAGAAAAGCGGCCATTTTCCACCATGATATTCGGCAAGCAGGCATCGCCATGGGTCACGACGAGA
TCCTCGCCGTCGGGCATGCTCGCCTTGAGCCTGGCGAACAGTTCGGCTGGCGCGAGCCCCTGAT
GCTCTTCGTCCAGATCATCCTGATCGACAAGACCGGCTTCCATCCGAGTACGTGCTCGCTCGATGC
GATGTTTCGCTTGGTGGTCGAATGGGCAGGTAGCCGGATCAAGCGTATGCAGCCGCCGCATTGCAT
CAGCCATGATGGATACTTTCTCGGCAGGAGCAAGGTGAGATGACAGGAGATCCTGCCCCGGCACTT
CGCCCAATAGCAGCCAGTCCCTTCCCGCTTCAGTGACAACGTCGAGCACAGCTGCGCAAGGAACG
CCCGTCGTGGCCAGCCACGATAGCCGCGCTGCCTCGTCTTGCAGTTCATTCAGGGCACCGGACAG
GTCGGTCTTGACAAAAAGAACCGGGCGCCCCTGCGCTGACAGCCGGAACACGGCGGCATCAGAG
CAGCCGATTGTCTGTTGTGCCCAGTCATAGCCGAATAGCCTCTCCACCCAAGCGGCCGGAGAACCT
GCGTGCAATCCATCTTGTTCAATCATGCGAAACGATCCTCATCCTGTCTCTTGATCAGAGCTTGATCC
CCTGCGCCATCAGATCCTTGGCGGCAAGAAAGCCATCCAGTTTACTTTGCAGGGCTTCCCAACCTT
ACCAGAGGGCGCCCCAGCTGGCAATTCCGGTTCGCTTGCTGTCCATAAAACCGCCCAGTCTAGCTA
TCGCCATGTAAGCCCACTGCAAGCTACCTGCTTTCTCTTTGCGCTTGCGTTTTCCCTTGTCCAGATA
GCCCAGTAGCTGACATTCATCCGGGGTCAGCACCGTTTCTGCGGACTGGCTTTCTACGTGAAAAGG
ATCTAGGTGAAGATCCTTTTTGATAATCTCATGCCTGACATTTATATTCCCCAGAACATCAGGTTAATG
GCGTTTTTGATGTCATTTTCGCGGTGGCTGAGATCAGCCACTTCTTCCCCGATAACGGAGACCGGC
ACACTGGCCATATCGGTGGTCATCATGCGCCAGC

1.2. Primers and gRNA sequences for recombination construction

Note: The bases with a red background indicate mutation sites, which prevent Cas9-mediated DNA cutting.

gRNA plasmid used: pU6-BbsI-chiRNA (Addgene No. 45946).

a. **For *dpp***

5'-Arm:

BamHI-Dpp-5'Arm-S: TTAGGATCCGTCGAGTTTGTCTTGGTTACGG

KpnI-Dpp-5'Arm-A: TTAGGTACCACCGCCCTCTCCGCCAGACA

3'-Arm:

BglII-Dpp-3'Arm-S: TTAAGATCTGGCAAGGGAGGACGGAATAAGCGACAGCCGAGAC

XhoI-Dpp-3'Arm-A: TTACTCGAGCGCAACGAAGTTTGAATACCC

Core *gRNA* sequence:

GCAAGGGCGGCCGGAACAAG

Note: The 20-nucleotide (nt) sequence inserted here can be referred to as the Core *gRNA* Sequence, as it represents the essential targeting region of the guide RNA.

b. For *dpp-TGKO*

KpnI-FRT insert S:**CGGAAGTTCCTATTC*tctagaaa*G*t*ATAGGAACTTCCGGTAC**

KpnI-FRT insert A:**CGGAAGTTCCTATaCtttctagaGAATAGGAACTTCCGGTAC**

BglII-FRT insert S:**GATCTGGAAGTTCCTATTC*tctagaaa*G*t*ATAGGAACTTCCA**

BglII-FRT insert A:**GATCTGGAAGTTCCTATaCtttctagaGAATAGGAACTTCCA**

Dpp-FRT-inner-3'Arm-LA:

TTCCTATaCtttctagaGAATAGGAACTTCATGGTGCGATGGTGGTGCG

Dpp-FRT-inner-3'Arm-RS:

TTCCTATTC*tctagaaa*G*t*ATAGGAACTTCACCACGCCATCCACTCAACC

Dpp-FRT-inner-5'Arm-RS:

TTCCTATTC*tctagaaa*G*t*ATAGGAACTTCGCTCACCCGCAATATCCTTC

Dpp-FRT-inner-5'Arm-LA:

CCTATaCtttctagaGAATAGGAACTTCTTGGAGCAAAGAAATCATAGGA

Note: We designed and generated two strains for *dpp-TGKO*:

Strain 1: KpnI site insert FRT / 3' Arm inner FRT

Strain 2: 5' Arm inner FRT / BglII site insert FRT

Development • Supplementary information

After testing, we found that the latter strain (Strain 2) functions effectively under Flp recombinase (flipase) mediation.The inner primers, designed to cooperate with the Arm-specific primers described in section 1.1, were fused together using the overlapping PCR method.

c. For *tkv*

5'-Arm:

KpnI-tkv-5'Arm-S: TTAGGTACCTCTCCCTGTCCGTGTTTATGC

KpnI-tkv-5'Arm-A: TTAGGTACCGACGATTTTGATGGGCACATCGATTAGACAG

3'-Arm:

BglII-tkv-3'Arm-S:

TTAAGATCTTAAGCAGCTGTTTGAAGTCTAGTTTTAAGTTTAACCTAAGT

SpeI-tkv-3'Arm-A: TTAACTAGTGTTACATCACCACCTCAACGC

gRNA sequence: （2 gRNA plasmids mixed together for injection）

1.GCTGTTTGTAGTCTCGTTTT

2.GCTGCTTAGACAATCTTAAT

d.For *sax*

5'-Arm:

KpnI-Sax-5'Arm-S: TTAGGTACCCGTAGCCATCGCAAGCGTCTG

KpnI-Sax-5'Arm-A: TTAGGTACCAACGCAGACTTCATCGAAGTCCAGGCGTATCTTC

3'-Arm:

BglII-Sax-3'Arm-S: TTAAGATCTTAGTGGTAGTAGTCGAGCCTTAGC

Sax-3'Arm-A: CCAGTTCTCTTTAGTCGGCAG

(An endogenous XhoI restriction site is present within the 3' Arm PCR product.)

gRNA sequence:

GATACGCCTGGACTTCGACG

e. For *punt*

5'-Arm:

KpnI-Punt-5'Arm-S: TTAGGTACCCTATAAGCAATTCGTAGATTCCTTG

KpnI-Punt-5'Arm-A: TTAGGTACCTAAGCAATTCGTAGATTCCTTGGC

3'-Arm:

BglII-Punt-3'Arm-S:

TTAAGATCTTAGAAGCTTTACTAAGCCACAGACCAGCCAGCGGATCT

BglII-Punt-3'Arm-A: TCAATGTCGGATTAGCAACTACC

gRNA sequence:

CTACGAATTGCTTATAGAAG

f.For *mad*

5'-Arm:

BamHI-Mad-5'Arm-S: TTAGGATCCGGCAGCCCGAAAACCAGCAC

KpnII-Mad-5'Arm-A: TTAGGTACCCATTCTGTTGCTGCTGCCGCTGATTGCTG

3'-Arm:

BamHI-Mad-3'Arm-S: TTAGGATCCGATACTGACGATGTGGAATCGAACACAAG

BamHI-Mad-3'Arm-A: TTAGGATCCAAATGGCGGAGTCGGATAGG

Development • Supplementary information

(BamHI enzyme cut and ligase in BglII clone sites in 3'Arm)

gRNA sequence:

GGTGTTCGATTCCACATCGT

g.For *babo*

5'-Arm:

BamHI-Babo-5Arm-S: TTAGGATCCGTTTGCCAAGGAGGATTCGG

BamHI-Babo-5Arm-A: TTAGGATCCCACGCTTATGCTAGCAAGTGTCTTTTTGATG

3'-Arm:

3Arm product: 1253 bp

BglII-Babo-3Arm-S: TTAAGATCTGAGGACAAGGTCAAGAACTGATTG

XhoI-Babo-3Arm-A: TTA CTCGAGTGTGACCCGGAATTAGCGC

(BamHI single wnzyme digestion and ligation into BglII cloning sites within the 3' Arm)

gRNA sequence:

GACACTTGCTAGCATCAGTG

1.3. Positive fly strains identification primers

sfGFP identification Primers (also used for YFP detection):

sfGFP-ID-S: GATGACGGCACCTACAAGACC

sfGFP-ID-A: CTCTGGGTGCTCAGGTAGTGG

mRFP identification primers:

mRFP-ID-S: CGAGGGCACCCAGACCGC

mRFP-ID-A: CGTCGGAGGGGAAGTTGG

tdTMT identification primers:

td-TMT-ID-5R: GAACTCTTTGATGACCTCCTCG

td-TMT-ID-3S: CCACCACCTGTTCCTGTACGG

Note: Due to the duplication of the tdTMT sequence, we determined that a pair of primers all in tdTMT sequence is ineffective. Therefore, the tdTMT primers (out forward direction) must be paired with another primer located in either the 5' Arm or the 3' Arm. These identification primers are also suitable for sequencing purposes.

YFP (Venus) identification primers:

YFP-Verus-ID-S: CAGTGCTTCGCCCGCTAC

YFP-Verus-ID-A: GATGGGGGTGTTCTGCTGG

1.4. Fly strain for transgenic docking

Strain: *y[1] M{nos-Cas9.P}ZH-2A w[*]* (Bloomington Stock Center, No. 54591)

2. Primers for *dpp-isoforms* identification

IsoID-dpp-RA-S: GAAGCCACAGCGAGATAGATAGG

IsoID-dpp-RA-A: GCCGCTCAGTTACGCTCCAA

IsoID-dpp-RB-S: AGTTTGGTTTCGTACTTGGCTCA

IsoID-dpp-RB-A: TCTGACACTCCTGAGAATTATGTATCC

IsoID-dpp-RC-S: ACGGCCAGCCAAAGCAAATA

IsoID-dpp-RC-A: CGGACGGTTCGCAGGTAATC

Development • Supplementary information

IsoID-dpp-RE-S: GCAGCGCACGTCGTCCAAC
IsoID-dpp-RE-A: CATGGGTGCAAGCGGGACG

Note: Relative Efficiency of the Four Primer Pairs:Dpp-RA: 0.99991 ± 0.00031, Dpp-RB: 0.99615 ± 0.00015, Dpp-RC: 1.00235 ± 0.00049, Dpp-RE: 1.00229 ± 0.00040

All four primer pairs were tested using an equal amount of DNA template, and no significant difference in amplification efficiency was observed among them.

3. Primers for mRNA *in situ* hybridization

Note: T3 RNA polymerase was used for sense probes generation, and T7 RNA polymerase was used for antisense probes generation.

T3-Prob-*dpp*-S:AATTAACCCTCACTAAAgggCAAGGAGGCGCTCATCAAG
T7-Prob-*dpp*-A:TAATACGACTCACTATAgggCACCAGCAGTCCGTAGTTGC
T3-Prob-*tdTMT*-S:AATTAACCCTCACTAAAgggCAAGGGCGAGGAGGTCATC
T7-Prob-*tdTMT*-A:TAATACGACTCACTATAgggCAGGGAGGAGTCCTGGGTC

4. *dpp-BRS1 to 4* segments related sequences

Note: *BMP-SE* Motifs:

Consensus Sequence: *GRCGNC(N)$_5$GTCT(G) (R = A/G; K = G/T)*

Color Coding: Yellow background base: *BMP-SE-like* partial sequence; Red background base: perfect *BMP-SE* sequence; Gray background base: spacer base

4.1 *dpp-BRS-1*:

ctcggggtttttgagcattgccgcctgtttgtaaatcaaagcgctatagcgagcgataagtggaaaatatgacgatcgaagct
actctaataaccgaaaaatccagggggtggggtgggggttgaagttttggtttggtatggggctatattgaatttctattcgtaga
cacaaaacgataaagctggtccgaaaagatcgtgtgtcagtgcaaaaggtgcaatgtccgcctgcgattgcaacacttgcc
gatgtgcaagtgagaggggtgggccgttagagcgagaggggagagcttgcggaggaggagagccaccatttttttagttgc
acctttcaccccgaagggccatccgtggtcgtaaaactttggccaggcgctctccggtctggtctggtctggtctgttcgtactg
ctccgctctctttttccctcaaatgggccaaaaggaggcgacgtcgctgccgcggtcgcagcgctgccgctgccgcagctac
cgccgctgcagacgtcgcttacctgccgaagaagaagagcagcgtTCAGTCGCGCAGCGCACGTCGTC
CAACGCACACACGCTCAGAGACACACCGACACGCACACAGATACAGATACGTTGAG
TCGCCGCCGCCGCGAAAGATACCAGATACTATCTGCCAGATACGAAGAGTTGGGCC
CTATAGTCGTCCCGCTTGCACCCATGGCCGCCTGAGTgtgagtgcaagagcggattggattgagt
ggaatacgaacgcgattccattccggtccacatccgaacccacatccgaatcctatccgaagccacctaacc

4.2 *dpp-BRS-2*:

cttttaatgcttatgcgggctgcccgctgtgttaattcgaattcaaactgtttccataaagtggagccatgtggctccctccccattt
cctacatggattgcttcacgccgctgacgagtgcgcgaagacttcattattatgcacaaagttatatatgtagtgtcctatgtatg
ggtatacaatgtcttaaacacaatcttcgttaacgtttaaagaaagtatagaaaacattagtaaataataaagcataactcga
acgcctcttgccatcaatcaagttaaggtaatagacttcagaacatcttttatgaaccacccattgcacttaattgcacttaatttg
gagctggcccctaggaaattacaattttcccaggggaattattaactggcgctaataaaacgcttcaaggtctcgggttaccat
cacagaatgcataaatcaagtgccgatcctgatggctaatggccggcgaaggaggaggctttgttaccaatttataaacaa
acaaaacgattccgacgacaacaaaggaagggacatcggcggcagcatcgaattaccacaagatcccggaccccga
accgaaaccaaaaccgaacccaagctaaaaacccagaccgaaacccatatccatgccccgcttcttacaaaacgttcag
gatcagctttcacaagatgcaacttagactttgagtccccagttgcagtttaccgccagtttgtcattcatcgcacagcgaagc
acacaataataacgttttaattgaacaaaagacacagtggagaatccagtgacaagaatccagggacccagtcgagcaa
caattgatggtcgtaggaattggaatccgattcggattcggattcggattgtatctgatggatggtgtttgtatttgttattgcagtttt

Development • Supplementary information

gcggctgcctcacgaggttcggtttctggttttcggttctcctgaagtgactgcgtctgtcacctttccatcaaactgaaggcagt
gaaaggctctcgacgaatgtccacgtgccaagcggcattgtttggcgacttttgttcgttttggctgcttctcgatggtaattgatt
agccggccagccgagaagaaacggagcgaatcaatggacacgtataaaggataacctaatgaggtccatttcttggttat
acccacaatgtgttgtgtgtgtgagtgtgtataaacagctgaaacactagaccacgtaataatagcaacataaggcctaattg
ggtgaccgaagttttcgggaaatagatatctttattaggacacccttatctctatcagtcatctatcatacgactacatttcatattt
gtgcctttatttagtaaaagatatatattgcatttaaagtaaaggagtttttcttggaaaatgacattgtataacaccatcaatgatc
aaagatagttactctcaagaccgtccatcttctagactttgattaaactatcctagctaagccaaggaacccgcattcttgcagt
tcccatccacttcgaggacaattaggactcacgcccccgttgcgtgacagggaaaacagaatgcgaaaagggatcggga
cgatcctgctgtgaccgaggtcctggcatgtggtcagtagaccgccgcctggtaccccgtccaggatcgggatgagaccca
ttcccatccagaactcgagagtttttgcgctgcgagatctttttgccaggcacttgcctttaacaaatgacacacgagcacttga
taaatgaacggcagctgataatggatttcgtatttggaattttggacgtcacttgttggtttccttggtccagtgaaagtagcgcca
gggatggcaagcggcgataagccgctcaaggactcagatttctggtggtccgataagctgggccgtgcgaaaggtaatta
agtgccattaaagttgagtggagagccggcattccgcagggccaatgtctctgtatctgtttatctgccggatgcagcggcag
agcgagagagcagaagagaccaaacgaatcgaaggaggagcacagaacgacactaacacaatcaccagcacaag
caccataa

4.3 *dpp-BRS-3*:

ttgacatgcgtgcgtctgtcagagcggcaattaaacaacatcaccggagtgccagttcggtattgggttcggtatcggaatag
ccatcgttgaaagggcaagcgctaatatttggtatttgtttgggaaccggcacccgaaatctgggaatccaggagctagcgc
caggccaccaacgtggctgctggaaaaccggtgaaaaccacagccggcgaactgtgaatgacgtccgaaaggagatc
atacttgtcaagagacaccattctagctcctggtaattagtcaactgaagtggccaagaagcgcgtcagtcgtcatccaaag
gcggtgctctttgaaagcaagtgaatataaaactaatttaaatttaaattgaatgtaacttttctttttaaaaagaaaaggtggtat
tcgggtggagatcccaagattcatatcaatttttccttataaacatataaaactataatacattgattgctaggaattgttcttagt
tttcgcaaaatggaatgcaggacctagaggatgggggccaacc**gacgcccgacagtct**ctccttctctcggaggagcag
tcataaatcaatgaggagcaggcagcaacatcgcaaccgcgcaacattgcaacattgcaacatcgcaagattgcagtattt
cagcatcgttgcaccgcattgcttctggccagagacaattggcatggctaatgcgaaaggcaactcataaatttattaattttta
attaaacttacaacgatttgtaattccaacatggtacctccatttatggcccagactgctgatgaccaacaactagttgagctgg
ccaactctgaatgaatctgcttcagctggagatggagctgtagctggagttgtagctggagctggagctggaggagctcattc
cgtcgatgtcagcccggaaacaactcattatcctaattaaaacgtgcgccatgcgcgcccgttgccatatatgagcaatgca
cgcgatctggcaatcgaggcgagtccgagtctgcggctgcatgcgaaattagaagattgccaagatccgagattcgagat
ggcacctccgataagatcggccatctggagctggagagccttgatttatcgccttgtattgttgtaagttttcagtatctgatggcc
aaggagagaatagagggttcgcttttagaagattgcaatactatataaatgcaagtgattttggatatttcgctactcttaataga
cctgaaggaattagtttgagagtttattaaagatttgtatttgtgtaccaatctgatatgctggtcattattataaggctctcacgctg
ccatctctgatcgcagagctgatgacgagcggctgtctcc

4.4 *dpp-BRS-4*:

Cgcaataagttcccaaaatcaccatcagcccaggtgggccaaaggcaaatgagacagtgggctaaagggttaaatactt
caacaagtaacattttaatatataaaaaaaatatttctatagagttttaatagctgttttacaagagtaagattgttggattaaatgt
atctttctatatatttatttatttgcatttattgtacattttcatcacactgtttttttgcattttcctcgctgtctaacctccagcgaattgagttt
ggaacttggccctctctggctgggaatctctcgatccgaaatcttctccaaaatgctgtgggtggtggggtcgggttgggttgg
gtggcattgggtctgggagtgggcagtcgacggggcctttacggtgaattgcgctgctcgtttttggcgcgtttgcgctttgcgat
taatgcgcttgtgtaaataaacaataaatattaagtgtgctacacgccgtgggcaaaacgacgacgaagaaaaggaaca
atctgttggcatcccccaccgccaggggcgatgctacttggggattgtagctggcgatggatcgctttggcgcgcaattaagt
gtaccactttatgcagggcacgatccaaagacaggaagaccccggataactccccacctgggtggggatggcatgcgtgt
ggtatcagggacgcggctaccctcgtcctcaccacctatcaccaaacgattttaagaggtcattggtcatttgtaaattgaactt

Development • Supplementary information

tggtattacattacatttctgtttagaaatgttcttattgattcagaattggctgtatgttaacctttcctgatccgcacgagctgcattt
ggctaacatgcccagttggctattgccggcgtaccgatcctaacaaatcttgataccctgcccccgatatatgcctcgctttcttc
cccagtctgccccactctctttcgtctgtcgtcgcatccaggcgtaacgcacaaattgtattgattatgcggctgccatgcactttt
cttgtatcttgtatctggtcgcgccgtaaatgttcatttgatttgcgaagcaatctgcttgttgtgtaataaattaatgcacttgatttca
acggttggtcaatgtctgcgatccggaggtgggtggcttccaccgaaggtgtgcgaagttccagggaaactcagccaatatc
cgacggattgaacagctgttgaactatggccagtcaggcgtgtggatgtgatctttaaagttcaaagggcattggctgtgatgc
cgttggatacattatacatatatttgggcatgcttctctgcttatgaaagtaaaatatctcttaagtgcccaatatgcgcatttaata
ttagtttatccccacttatcacctctgctgaccttgacttactctttcgctgtgttagccataactcactcacttcggatggatggatat
attttctcggcaactgtttacatgtacatttagttaaatgcaattcggtccataaatttcgcgtgttctacccggaatcggttcattag
ataatagttttaatttgcttatataatgcttatataagcacgctggattaaggcgcactgagtaccgcgaaaagcaatcaaagg
tcagtcgaaaaaatacagacactttgaaggcgccaacaacgcagaaaagcgtggcggcaacttgttcctcgttgtttcgtac
ttagt

5.Primers for construct of *luciferase* assay

SbfI-BRS-luc-1S: TTACCTGCAGGCTCGGGGTTTTTGAGCATTG
NotI-BRS-luc-1A: TTAGCGGCCGCGGTTAGGTGGCTTCGGATAGGA
SbfI-BRS-luc-2S: TTACCTGCAGGCTTTTAATGCTTATGCGGGCTG
NotI-BRS-luc-2A: TTAGCGGCCGCTTATGGTGCTTGTGCTGGTGA
SbfI-BRS-luc-3S: TTACCTGCAGGGAATCTGTATCGCTATCGCTGTCTC
NotI-BRS-luc-3A: TTAGCGGCCGCGGAGACAGCCGCTCGTCATCAG
SbfI-BRS-luc-4S: TTACCTGCAGGCGCAATAAGTTCCCAAAATCAC
NotI-BRS-luc-4A: TTAGCGGCCGCACTAAGTACGAAACAACGAGG

6.Primers for *lacZ* reporter for cell culturing applications

6.1. Primers for *wg*-promoter cloning:

EcoI-Wg-promoter-S: TTGAATTCCGATTGTGAGCGATTAGCAGGG
BglII-Wg-promoter-A: TTAGATCTGATAGAATACACTCGGCTCGCTC

6.2. Core sequences for peaks *a* to *t* , for 200 bp in length. For motifs scanning in MEME with XSTREME program.

>a
atcaccagcacaagcaccataaccatctcaagaacagcagaacagcagagcacacaaagacagaacgacggacaaatcagacagacga
ttaacatacaaaaggatctgcacgaactttggagcccgccttcttcgcggcaaatgtatctgtatctgtttctgtatctgtgtggagtgctctggagtttttc
tgtgcgcca
>b
agtagggaggcagaagagccggacagacggcgacaagtgaagaactggacaggaacaaaggctacttaagcagccttggcgccagttga
ccatgtgtggcgaactaaagtaaagtgatgtacattgaagtatagccacaatgtctggcaatgctccatttgctcagtctttcgaccagacgacgcc
aatggtcttcaatg
>c
acaacaaaaggcagttaaaatgcatttagctggcagtgtatgtgtgtgtatctgtgtgcctgcgtgcgaaagagctggcatgtgtcattcttatggctg
ccaccgcggcccttttttttacctcttgccttccgaccaataaaattatactaattcctgactttttgatttcattttctgtttgtgttgttgccaaggat
>d
tgtgcggtggatcggtgatcatgatcgtgatcatcgtcattataccatcgctgcctggctggctggcacatctgaacatccgagcatcggaacaactg
aacttctgagcatccgaggtcccagactccaggaatgcgcagacAGTTTGGTTTCGTACTTGGCTCATTGCGCTCGTGC
AGCTCGATATCCCAATCCCCGAG
>e
ctgggaatccgagtccgagaaacgctagacgagatgcgtgatcatgatcgcgataatgatggagatggggctaaagccccaactttccgagttct
cctctcccgatcggcgactcgactctctcggctacgacggacctctctctcgtggagtggagctgccgaagtgtgtatctgtgtgctcgccgcgccg
atgtatct

Development • Supplementary information

>f
AGCCACTTGGCGTGAACCCAAAGCTTTCGAGGAAAATTCTCGGACCCCCATATACAAATATCGGAAA
AAGTATCGAACAGTTTCGCGACGCGAAGCGTTAAGATCGCCAAAAGATCTCCGTGCGGAAACAAAG
AAATTGAGGCACTATTAAGAGATTGTTGTTGTGCGCGAGTGTGTGTCTTCAGCTGGGTGTGTGGAAT
>g
GATGCCCCCAGAAACAATTCAATTGCAAATATAGTGCGTTGCGCGAGTGCCAGTGGAAAAATATGTG
GATTACCTGCGAACCGTCCGCCCAAGGAGCCGCCGGGTGACAGGTGTATCCCCCAGGATACCAAC
CCGAGCCCAGACCGAGATCCACATCCAGATCCCGACCGCAGGGTGCCAGTGTGTCATGTGCCGCG
GCA
>h
gtgagaactgcacacaaaacccttgcataatggcagtcgtgcattcgatatcgagccaagttcatttgatattgaaaaatatggtacttgtcgatccg
aggaattttcgggttgttcgccttgtcgatggcatgtggcattcagtcgtgtcattcgagagaactcaaattgtccataggaattggtaatgggatatattt
>i
ttttccctcaaatgggccaaaaggaggcgacgtcgctgccgcggtcgcagcgctgccgctgccgcagctaccgccgctgcagacgtcgcttacct
gccgaagaagaagagcagcgtTCAGTCGCGCAGCGCACGTCGTCCAACGCACACACGCTCAGAGACACAC
CGACACGCACACAGATACAGATACGTTGAGTCGCC
>j
CACCGACACGCACACAGATACAGATACGTTGAGTCGCCGCCGCCGCGAAAGATACCAGATACTATC
TGCCAGATACGAAGAGTTGGGCCCTATAGTCGTCCCGCTTGCACCCATGGCCGCCTGAGTgtgagtgc
aagagcggattggattgagtggaatacgaacgcgattccattccggtccacatccgaacccacatc
>k
gcaacaaagttggcaatatcgtgtcgagtttagcgcccgcctttgtttggattggagtgatcgctagatcgcctattttatggtgcttttgattgggccag
agcatggcaaatgtaacataactttatggatctgccgatgtttactctgctcggaatcgtgggcatctcttagataaaaaaggacgaaagtaggaag
ta
>l
tgtggtcagtagaccgccgcctggtaccccgtccaggatcgggatgagacccattcccatccagaactcgagagtttttgcgctgcgagatctttttg
ccaggcacttgcctttaacaaatgacacacgagcacttgataaatgaacggcagctgataatggatttcgtatttggaattttggacgtcacttgttgg
ttt
>m
aataaaaatgtgtcaatttgcgaaacatttcggagtcgttggtgatgcgacaaaaagctgaaatttgaactttagcaggcgcttgcgacttttcacgg
ctcgaattgagctgagttcgagttatagacatagtatcgtatctggtatctggtatctggtgcgcgtagtttcactgaatgggcggcgagcttggaaatt
cc
>n
caacgagagtacctctcagtgcccctgtatttgtgggatatgtaccagccagcatttgatttatgctcaactttgatatttaccgcctaataaacaaaaa
attaacgcaaacgcagctagccgcattatagatgtatctatgtatctgaatctgtatcgctatcgctgtctctatctccatctgtatctgtatctatttg
>o
tcgtgtgtcagtgcaaaaggtgcaatgtccgcctgcgattgcaacacttgccgatgtgcaagtgagaggggtgggccgttagagcgagagggga
gagcttgcggaggaggagagccaccatttttagttgcacctttcaccccgaagggccatccgtggtcgtaaaactttggccaggcgctctccggtc
tggtctggt
>p
ggcccagctcccttgttgccccagccccagcttcagtccacagttgcattgtaaattcgtaaaacaataaagaaataaattactttgggccccaagt
ccaagctcaagaactcttgtccaggccgggggaaccacagatacagactcaccgactcttcagctcaccagctcacaggctcacaaagaaca
cgacaaagcgt
>q
acttaagagcgttcagaaattattccgtcaaaatgaggagacttcccaaaaagcgaaactggccaacacccgaagtgtctatgcggacacctca
gcaacatcctatgcccagctccgatccgatctgctcctctgtcgagttgaagttgaaacaaagatataaaaataaaaacaaaaatcatataaaaa
atcgttaataa
>r
Tcataataagcccaaaaacagcaaacaaagctggccaaattcggagtgctgggggcgattcgctgggccgtctcaatagttccttttattattaata
aataaggcttagtgggtctgtttgttgtcgttgctgctgccagtcggtctgcttgtattttattatactgctggctgagttctgttctcttctgttggtgcta
>s
ttaagatagctatggaaagtgaaaagatgaagctatgaaacttagggtgtcccttggggttaaatgaagttttattgtacatatttttctatgaatctctca
tacctctttgtagggtattgcaaagagttgttcaaacaattgtctgctgcatgtctagtcgtcatagtcatcgtcatcgtcatcgccatcgccatcga
>t
GGTGGCAGGATGGCCTGACCAGGAGATATCCCCGCGTCCACATCGCAGACCCTTATCAGTGAGTG
GCGATCGGCTCTTGGACGGTGATAATGACTTGTAAGTACAAAATGGGTTGTCAGGATGGTTGCTGCT
GCTGCAGATGTTGCTGCTGCAGATGCTGCTGCGGTTCTTGCGGCAATCTTCTGCTGCACGCTGCAA
GC

Development • Supplementary information

6.3. Primers for cloning fragments *ab* to *o*:

ab-BglII-*lacZ*-S: TTAGATCTGAAAGTAGCGCCAGGGATGG
ab-XhoI-*lacZ*-A: TTCTCGAGTTGGTAGGTGGAGCTGGCTTG
cd-BglII-*lacZ*-S: TTAGATCTCATTATCCGTCGTCTTCTTGGC
cd-XhoI-*lacZ*-A:TTCTCGAGTCAGCCGAGTCGCTTCCTTC
egf-XhoI-*lacZ*-S: TTCTCGAGCCACAAGCCGAACCTCAAAG
egf-KpnI-*lacZ*-A:TTGGTACCTCCGATAATTGTAGATGAAGTAGAT
ij-BglII-*lacZ*-S: TTAGATCTCTTTTTCCCTCAAATGGGC
ij-XhoI-*lacZ*-A:TTCTCGAGCGGAATATGAGCGGCAACG
k-BglII-*lacZ*-S: TTAGATCTCTGATTGCGTGTTGTTATGTTCC
k-KpnI-*lacZ*-A:TTGGTACCTTGGCGTTATCTTCTGGTCTTGT
l-NotI-*lacZ*-S:TTGCGGCCGCCGCATTCTTGCAGTTCCC
l-XbaI-*lacZ*-A:TTTCTAGATTATCGCCGCTTGCCATCCC
m-XhoI-*lacZ*-S: TTCTCGAGTGCGTGAGTGTCCAAGGGTT
n-BglII-*lacZ*-S:TTAGATCTGAACCCAGAGCCAAGTGAGCG
n-KpnI-*lacZ*-A:TTGGTACCCTTTCAACGATGGCTATTCC
o-XhoI-*lacZ*-S: TTCTCGAGTTTTTGAGCATTGCCGCCTGT
o-KpnI-*lacZ*-A:TTGGTACCGGAGCAGTACGAACAGACCAG

6.4 RT-qPCR primers for *lacZ* mRNA detection

RT-RPL32(rp49)-S: CAAGCACTTCATCCGCCACC
RT-RPL32(rp49)-R: CGATCTCGCCGCAGTAAACG
RT-*lacZ*-S: GCTGGCGTAATAGCGAAGAGG
RT-*lacZ*-A: GGATAGGTCACGTTGGTGTAGATG

7.Primers and 20nt core sequences for constructs of *UAS-CRISPR/Cas9-dual-gRNAs* generation in transgenetic flies

gRNA-up:
TAgaagacaccttcGNNNNNNNNNNNNNNNNNNNGTTTTAGAGCTAGAAATAGCAAGT

gRNA-down: (core sequence reversed)
TAgaagacacaaacNNNNNNNNNNNNNNNNNNNNCGAAGTATTGAGGAAAACATACCTAT

BMP-GFP-up: GGTGGTGCAGATGAACTTCA
BMP-GFP-down: CAACCCCAGAAACGAGTCCC
BMP-SE1-up: GTTACGCATCGCAGACTAAG
BMP-SE1-down: CCCCTGAAAGCATGGCTCGC
BMP-SE1us-down: CTTAAGCGGTCGGAATCTTA
BMP-SE2-up: GGTCTGTGGAAACTGCCGTT
BMP-SE2-down: CGAGTTGAACCAGACTTCGG
BMP-SE3-up: GCGAGCACGATTTGCTTGTA
BMP-SE3-down: CACCGACGTCTAGGAGAGTA
BMP-SE4-up: GTGCAGGACCTAGAGGATGG
BMP-SE4-down: CACTGACGAGGAGGCTCTCT

Development • Supplementary information
